# Genetic variability in the rat Aplec C-type lectin gene cluster regulates lymphocyte trafficking and motor neuron survival after traumatic nerve root injury

**DOI:** 10.1186/1742-2094-10-60

**Published:** 2013-05-08

**Authors:** Rickard PF Lindblom, Shahin Aeinehband, Roham Parsa, Mikael Ström, Faiez Al Nimer, Xing-Mei Zhang, Cecilia A Dominguez, Sevasti Flytzani, Margarita Diez, Fredrik Piehl

**Affiliations:** 1 Department of Clinical Neuroscience, Unit for Neuroimmunology, Karolinska Institutet, Stockholm, Sweden; 2 Neuroimmunology Unit, CMM L8:04, Karolinska University Hospital, 171 76, Stockholm, Sweden

**Keywords:** Neuroinflammation, Neurodegeneration, Microglia, Aplec

## Abstract

**Background:**

C-type lectin (CLEC) receptors are important for initiating and shaping immune responses; however, their role in inflammatory reactions in the central nervous system after traumatic injuries is not known. The antigen-presenting lectin-like receptor gene complex (Aplec) contains a few CLEC genes, which differ genetically among inbred rat strains. It was originally thought to be a region that regulates susceptibility to autoimmune arthritis, autoimmune neuroinflammation and infection.

**Methods:**

The inbred rat strains DA and PVG differ substantially in degree of spinal cord motor neuron death following ventral root avulsion (VRA), which is a reproducible model of localized nerve root injury. A large F2 (DAxPVG) intercross was bred and genotyped after which global expressional profiling was performed on spinal cords from F2 rats subjected to VRA. A congenic strain, Aplec, created by transferring a small PVG segment containing only seven genes, all C-type lectins, ontoDA background, was used for further experiments together with the parental strains.

**Results:**

Global expressional profiling of F2 (DAxPVG) spinal cords after VRA and genome-wide eQTL mapping identified a strong *cis*-regulated difference in the expression of Clec4a3 (Dcir3), a C-type lectin gene that is a part of the Aplec cluster. Second, we demonstrate significantly improved motor neuron survival and also increased T-cell infiltration into the spinal cord of congenic rats carrying Aplec from PVG on DA background compared to the parental DA strain. *In vitro* studies demonstrate that the Aplec genes are expressed on microglia and upregulated upon inflammatory stimuli. However, there were no differences in expression of general microglial activation markers between Aplec and parental DA rats, suggesting that the Aplec genes are involved in the signaling events rather than the primary activation of microglia occurring upon nerve root injury.

**Conclusions:**

In summary, we demonstrate that a genetic variation in Aplec occurring among inbred strains regulates both survival of axotomized motor neurons and the degree of lymphocyte infiltration. These results demonstrate a hitherto unknown role for CLECs for intercellular communication that occurs after damage to the nervous system, which is relevant for neuronal survival.

## Introduction

Both innate and adaptive immune responses, involving local as well as blood-derived immune cells, have been shown to exert both protective and detrimental effects after traumatic nerve injuries, but there is still limited knowledge on how this regulation is executed.

Probably the most studied immune cells, with regard to traumatically induced immune responses, are the microglia [1], which are a versatile and heterogeneous cell population. Microglia also constitute the main antigen-presenting cells of the central nervous system (CNS) [2], and are as such key players in eliciting subsequent immune responses, which include recruitment of immune cells both from the innate and adaptive immune compartments.

Innate immune cells, like neutrophils [3], monocytes and macrophages [4], infiltrate the CNS after acute injuries. Whereas neutrophils have mostly been linked to unfavorable consequences [5], monocytes and macrophages have been associated both with protection and exaggeration of injury [6,7]. Regarding the role of cells from the adaptive immune system following traumatic CNS injuries, both T and B cells have been shown to confer both protective and deleterious effects [8-10].

These partly discordant results likely depend on the complex interplay between the differentcelltypes and the milieu, which in turn may differ depending on genetic background. An interesting example of the synergy between immune cells in the CNS was recently demonstrated in a mouse model of amyotrophic lateral sclerosis (ALS), where T cells in concert with microglia were shown to provide significant protection from motor neuron loss [11]. In this model, upregulation of CD69 on CD3+ T cells and an altered microglia phenotype with upregulated expression of dendritic cell (DC) receptors conferred the observed protection [11]. CD69, also known as Clec2c, is commonly used as an early marker of T-cell activation [12] and is a C-type lectin (CLEC) receptor, believed to be important in modifying immune responses, for example by downregulating detrimental Th17 responses [13].

CLECs are otherwise mostly studied in the context of dendritic cells and are thought to be involved in initiating and shaping immune responses [14]. The CLEC family includes a large number of molecules, most of which have unknown binding partners and functional roles, but are nevertheless thought to be important for innate immune reactions [15,16]. Structurally, the CLECs display certain similarities to some of the complement proteins [17], which on the other hand are well established as involved in neuroimmune responses [18].

On rat chromosome 4 (mouse chromosome 6 and human 12), a set of CLECs is grouped together and forms a gene cluster called the antigen-presenting lectin-like receptor gene complex (Aplec). A naturally occurring variability in the Aplec cluster was originally positioned to regulate susceptibility to experimental autoimmune arthritis in rats [19]. Subsequent studies using a congenic strain with Aplec from the PVG rat ontoDA background demonstrated regulatory influence of this region for susceptibility to experimental allergic encephalomyelitis (EAE) (unpublished data) as well as the response of macrophages to infectious stimuli [20]. In humans, the corresponding region shows a suggestive association with rheumatoid arthritis [21-23]. Taken together these observations suggest this gene cluster is of importance for regulating immune responses involving both the adaptive and innate arms of the immune system. However, the exact molecular pathways for the disease regulatory effect are still unknown and the C-type lectins have so far not been studied in the context of traumatic nerve injuries.

The inbred rat strains DA and PVG differ substantially in degree of spinal cord motor neuron death following ventral root avulsion (VRA), which is a reproducible model of localized nerve root injury that induces cell death and localized inflammation in the spinal cord. An unbiased global expression profiling study in a large F2 (DAxPVG) intercross subjected to VRA revealed strong differences in expression of a gene in the Aplec cluster. Subsequent experiments with congenic Aplec rats demonstrated a significantly improved survival of avulsed motor neurons, which was associated with increased T-cell infiltration, compared to the parental DA strain. These observations suggest that the Aplec region is important for inflammatory activation occurring after nerve root injury, which is relevant for nerve cell survival.

## Materials and methods

### Animals and surgery

All animal experiments were approved by the local animal ethics committee, Stockholms Norra Djurförsöksetiska Nämnd, under the ethical permit numbers N122/11 and N478/12.

The DA.RT1^*av1*^, hereafter called DA, strain was originally obtained from Professor Hans Hedrich (Medizinische Hochschule, Hannover, Germany), whilst the PVG.RT1^*av1*^ strain, hereafter called PVG, is a major histocompatibility complex (MHC) congenic strain originating from Harlan UK Ltd (Blackthorn, UK). Both are kept as breeding colonies at our in-house animal facility. The Aplec strain, generously provided by Dr Jian Ping Guo, was created by repeated backcrossing of DAxPVG F1 animals onto DA background as previously described [19,22,24]. The congenic PVG insert spans from 159.46 to 159.98 Mb on chromosome 4. It contains six characterized genes and one uncharacterized gene, all C-type lectins, according to the latest version of the Ensembl database [25]. Animals were bred under pathogen-free and climate-controlled conditions with 12 h light/dark cycles. They were housed in polystyrene cages with wood shavings and provided with a standard rodent diet and water *ad libitum*. The F2 (DAxPVG) intercross used for expressional profiling has been previously described [26]: in brief breeding couples composed of either a DA male and PVG female, or a DA female and a PVG male were used to generate two groups of offspring (F1), which were subsequently mated reciprocally, to generate four groups of F2 progeny. A total of 144 F2 animals, equal numbers from all four groups with both female and male rats were included.

All animals except for naïve controls were subjected to a unilateral avulsion of the left L3-L5 ventral roots at an age of 9 to 10 weeks. This procedure was done under deep isoflurane anesthesia. In brief, a dorsal laminectomy was performed at the level of the L2-L3 vertebrae. The dura was carefully opened with the point of a needle. By gently moving the sensory nerve roots, the L3-L5 ventral roots could be identified and avulsed from the spinal cord using a micro-forceps. The muscles and skin were then closed in layers and the animals were allowed to recover. Post-operative analgesia (buprenorphine, 0.1 ml, 0.3 mg/ml, RB Pharmaceuticals, Slough, UK) was given subcutaneously twice daily. Five- or 21-day post-operative survival was used throughout. Eight DA and eight Aplec rats were used in the neuronal count experiment. Nine DA and nine Aplec rats were used in the microarray studies. Five DA, five PVG and five Aplec rats were used for flow cytometry experiments. All animals were euthanized with CO_2_ and perfused via the ascending aorta with 200 ml PBS containing heparin 10 IE/ml (Leo Pharma AB, Malmö, Sweden) to ensure removal of as much blood as possible from tissues. All spinal cords were examined in a dissection microscope to verify completeness of the avulsion injury at the correct three levels and animals with incomplete lesions were excluded from the study; for this reason six F2 animals, one DA and one Aplec, both from the neuronal count experiments, were excluded. For the RT-PCR experiments, the ipsilateral ventral quadrant of the L3 segment was dissected, with careful removal of the scar on the ventral surface of the spinal cord, snap frozen and stored at −70°C. The L4 and L5 cord was dissected *en bloc* and stored at −70°C until cryostat sectioning. For the flow cytometry experiments the ipsilateral side of the L3-5 segments was taken *en bloc* for further processing.

### Myelin oligodendrocyte glycoprotein immunization and T-cell sorting from lymph nodes

Five DA and five Aplec female rats, 8 to 10 week old, were immunized using recombinant myelin oligodendrocyte glycoprotein (rMOG), aa 1–125, from the N-terminus, which was first expressed in *Escherichia coli* and purified to homogeneity by chelate chromatography, as previously described [27]. The purified protein, dissolved in 6 M urea, was dialyzed against PBS. The rats were anesthetized with isofluorane (Forene, Abbott Laboratories, Abbot Park, IL) and immunized with a single subcutaneous injection at the dorsal tail base with 200 μl of inoculum containing rMOG (12.5 μg/rat) in saline emulsified in a 1:1 ratio with incomplete Freund’s adjuvant (IFA) (Sigma Aldrich, St. Louis, MO). At 7 days after immunization, before clinical EAE had developed, animals were euthanized using CO_2._ Draining inguinal lymph nodes were collected from the ten immunized rats, and also ten naïve animals, five DA and five Aplec females. The lymph nodes were placed in DMEM (Gibco-BRL, Grand Island, NY), enriched with 10% fetal calf serum, 1% L-glutamine, 1% penicillin-streptomycin and 1% pyruvic acid (all from Life Technologies, Paisley, UK) before being mechanically separated by passing through a mesh screen with the bolus of a syringe. Cells were stained for 30 min at 4°C with CD3, CD45RA and RT1B antibodies (all from BD Biosciences, San Jose, CA, USA), and sorted with a MoFlo cell sorter (Beckman-Coulter, Brea, CA). After sorting, total RNA was isolated from the T-cell subset using a standard protocol (see below, RT-PCR section) before being converted into cDNA (also below).

### RT-PCR

Spinal cord samples were dissociated in Lysing Matrix D tubes (MP Biomedicals, Irvine, CA) on a FastPrep homogenizer (MP Biomedicals, Solon, OH) and resuspended in RLT buffer (Qiagen, Hilden, Germany) for total RNA preparation. Cells (T cells and glia) were lysed directly in the RLT buffer. Total RNA was extracted, purified and on column DNase I treated using an RNeasy Mini kit (Qiagen) and RNase-Free DNase Set (Qiagen), according to the manufacturers’ protocols. RNA from the L3 segments was further processed for cDNA preparation as described below, and RNA from the L4 segments was taken for array hybridization, as described below. All steps were performed under RNase-free conditions. Real-time PCR was conducted using a three-step PCR protocol using IQ5 or the Bio-Rad CFX 384 SYBR green optical system (Bio-Rad, Hercules, CA). All primers and probes were designed with Beacon Designer 5.0 software (Bio-Rad), and tested for specificity by running the amplified product on gels with silver staining. Two house-keeping genes were used to normalize the levels of mRNA expression of the studied transcripts; hypoxanthine guanine phosphoribosyl transferase (HPRT) and glyceraldehyde 3-phosphate dehydrogenase (GAPDH) and normalized expression levels were calculated with the IQ5 software or the Bio-Rad CFX manager v1.6 (Bio-Rad). For primer sequences see Table 1.

**Table 1:** Sequences of primers used for RT-PCR

**Primer name**	**Forward sequence**	**Reverse sequence**
Gapdh	TCAACTACATGGTCTACATGTTCCAG	TCCCATTCTCAGCCTTGACTG
Hprt	CTCATGGACTGATTATGGACAGGAC	GCAGGTCAGCAAAGAACTTATAGCC
Cd11b	ATCCGTAAAGTAGTGAGAGAAC	TCTGCCTCAGGAATGACATC
Gfap	AAGCACGAGGCTAATGACTATCG	AAGGACTCGTTCGTGCCG
Mrf-1	GGAGGCCTTCAAGACGAAGTAC	AGCATTCGCTTCAAGGACATAATA
Cd4	GCTCCCACTCACCCTTCAGATAC	CTTCACCTTCACTCAGTAGACATTGC
Cd8a	ACACCAGAGATAGTCCCAGTTTCAG	GCCAGCAATTTCCCAGTTCCTTAC
Cd69	ACGCTACCCTTGCTGTTATTGATTC	GTCTTCTTCCTTGTGTTCCATAGTCC
Clecsf6/Dcir1	CTGAACCGTGATGCTGCTTA	TGCTGTTTACCACTGCAAGG
Clec4a2/Dcir2	CCATCATCCAAGTAAGCCAGGTTC	GAGTCAGTTGAAGTAAAGTAGCAGTAGG
Clec4a3/Dcir3	TGCCACAAGTTCTTCAAGG	TCCAATTCAGTATAGTTCAGTTCC
Clec4a1/Dcir4	CATTCGTCCGTGGAAGACAAA	TGCAGAGTCCCTGGAAGTGAA
Clec4b2/Dcar1	TGCTCATCTGTTGGTGATCCA	TGTAAAATAACCCCAACGAGTGTCTA
Clec4e/Mincle	TTTCACAGAGTCCCTGAGCTTCT	TCCCTCATGGTGGCACAGT
Clec4d/Mcl	CACAAGGCTAACATGCATCCTAGA	GCAAAGTAACAGTTAGACTGGAATGCT

RT-PCR: real-time polymerase chain reaction.

### Microarray expressional profiling

RNA from the L4 spinal cord segment from five naïve DA and five naïve Aplec rats, as well as from four DA and four Aplec rats 5 days post-VRA operation, was sampled for global expressional profiling. The microarray analysis was performed at the Bioinformatics and Expression Analysis Core Facility (BEA) facility of the Karolinska Institute using Affymetrix Rat gene 1.0 ST Array chips (Affymetrix, Santa Clara, CA). In the first-round synthesis of double-stranded cDNA, 100 ng of total RNA was used. RNA was reverse transcribed using a whole transcript cDNA synthesis and amplification kit (Affymetrix UK Ltd, High Wycombe, UK). The resulting biotin-labeled cRNA was purified using an *in vitro* transcription (IVT) clean-up kit (Affymetrix) and quantified using a NanoDrop ND-1000 Spectrophotometer (A260/280; NanoDrop Technologies, Wilmington, DE). An aliquot (5.5 μg) of cRNA was fragmented by heat and ion-mediated hydrolysis at 94°C for 35 min. Confirmation of RNA quality was assessed using the Agilent 2100 Bioanalyzer (Agilent Technologies, Santa Clara, CA). Target labeling, and array hybridization, washing and staining were performed as described in the GeneChip Whole Transcript (WT) Sense Target Labelling manual [28]. Arrays were scanned using the GeneChip Scanner 3000 7G and the GeneChip Command Console (Affymetrix) with default set-up. For each sample, 2 μg of total RNA was used. Probe details can be found on the Affymetrix website [28]. The microarray data is available in Minimal Information About a Microarray Experiment (MIAME) compliant format at the ArrayExpress Database [29] under accession code E-MTAB-920. The expression data was imported into the software Partek Express (Partek Incorporated, St Louis, MO), where it is normalized using the RMA algorithm [30]. On the Rat gene ST 1.0, array genes are represented by multiple probe sets. Affymetrix assigns these probe sets to transcript clusters. Briefly, raw expression intensities are background corrected, quantile normalized, log2 transformed and summarized at a probe set level. The resulting normalized data was then filtered using the following criteria; first all genes significantly differing in expression between the operated DA and Aplec rats were selected (*P* < 0.01 was chosen as the cut-off). Second, the identified genes were checked to see if they were different between naïve Aplec and operated Aplec rats, with *P* < 0.01, which they all were, to ensure that the identified genes shown to differ in injured DA and Aplec rats indeed were injury related and not already different in the naïve state. The resulting gene list was then imported into the DAVID database to investigate for potential pathway enrichment [31,32].

### Expression quantitative trait loci (eQTL) mapping in the F2 (DAxPVG) intercross

The main analysis of the microarray data from the F2 intercross will be published separately (unpublished data), but the expression data is available in MIAME-compliant format at the ArrayExpress Database [29], accession code E-MTAB-303. Raw expression intensities were background corrected, normalized, log2 transformed and summarized on a probe set level as above but then instead implemented in the Bioconductor package oligo. Average expression values of all probe sets annotated to a transcript cluster were used to measure expression on the level of a gene based on annotation from Bioconductor package pd.ragene.1.0.st.v1.

The genotyping of the F2 intercross has been previously described (unpublished and [26]). Briefly, genomic DNA was extracted from rat tail tips according to a standard protocol [33]. Polymorphic microsatellite markers were selected from the Rat Genome Database [34] and the Ensembl database [25]. The F2 intercross was genotyped with 113 microsatellite markers evenly distributed across the genome, with an average distance of 20 cM based on previous knowledge of optimum spacing [35]. The successful genotyping rate was 95.3%. Subsequently, we mapped eQTLs for all transcript clusters using the QTL reaper software [36] against the 113 genomic markers. In order to assess the genome-wide significance of eQTLs we performed 10^6^ permutations: *P* < 0.01 at a genome-wide level was considered significant. We classified eQTLs into *cis*- or *trans*-acting according to the distance between the locations of genetic marker and the affected transcript. If the distance was smaller than 20 Mb we assumed *cis*- and otherwise *trans*-regulation.

### Flow cytometry

The L3-5 segments of the ipsilateral side of the spinal cords sampled at 5 days after VRA were homogenized in 4 ml 50% Percoll solution (Sigma-Aldrich, St Louis, USA) and transferred to a sterile 15 ml Falcon tube (Sarstedt, Nümbrech, Germany). A density gradient was made by carefully adding a bottom layer of 3 ml 63% Percoll solution (Sigma) under the middle layer with the cell suspension in 50% Percoll and finally adding a top layer of 30% Percoll. All Percoll solutions were made by diluting adding Percoll in 1xHBSS (Hank’s balanced salt solution) (Invitrogen, Sweden) and adding 0.1% glucose and 0.1% BSA. After centrifugation at 1000*g* at 4°C for 30 min without a break, the cells were collected, washed at 600 *g* at 10°C for 15 min with PBS containing 5% FCS and 0.01% tris-EDTA (Tris(hydroxymethyl)aminomethane (Tris)- Ethylenediaminetetraacetic acid (EDTA)), stained and resuspended in 100 μl of PBS with 0.01% tris-EDTA for flow cytometry. Cells were stained for 20 min at 4°C using the antibodies CD3-APC and CD11b-FITC (both from BD Biosciences, San Jose, CA), then washed in PBS before staining. They were visualized on a Gallios flow cytometer (Beckman-Coulter) with Gallios software and analyzed using Kaluza v.1.0 (both Beckman-Coulter).

The cells from each sample were acquired under a constant speed, but with acquisition times that varied between the samples because of small differences in cell density, with the acquisition stopping when a fixed number of cells (10,000) were counted. In order to normalize the samples and get a more representative number of the total number of cells per gate per sample, the number of cells per each specific gate was divided by the acquisition time for that specific sample, and presented as the number of cells per 100 seconds.

### Nerve cell counts

Serial transverse frozen sections (14 μm) of tissue from blocks containing the spinal cord L4-5 segments from DA and Aplec animals 21 days post-VRA were cut in a cryostat, starting from the mid L5 segment and serially cutting towards the rostral end of the L4 segment. The spinal cords were mixed randomly in each block used for consecutive sectioning and mounting on tissue slides. Nerve cell counts of cresyl violet counterstained sections were performed as described previously [37,38]. In brief, motor neurons with a visible nucleus were counted by a single, blinded observer. Every fifth spinal cord section, with a total of 25 sections from each rat, was counted. Each slide contained sections mounted in a similar fashion in order to reduce the risk of bias. Shrinkage of cell bodies of axotomized nerve cells occurs following VRA; however, we have previously found that the degree of shrinkage does not differ in a panel of inbred strains that included DA and PVG [38]. No correction for cell shrinkage was performed here as this would not affect the relative difference in nerve cell survival between the two strains. The degree of neurodegeneration is presented as a ratio of the total number of motor neurons on lesioned and unlesioned sides, respectively, in each rat.

### Immunohistochemistry

Spinal cord sections were serially cut (14 μm) on a cryostat (Leica Microsystems, Wetzlar, Germany) at the level of the L4 segment, thawed onto Superfrost plus microscope slides (Menzel-Gläser, Braunschweig, Germany) and stored at −20°C until further processing for immunohistochemistry. Sections were post-fixed in RT 4% formaldehyde (Iba1) for 30 min or ice-cold acetone (Gfap) for 10 min, rinsed in PBS and incubated overnight at 4°C with primary antisera directed against Gfap (rabbit anti-human, 1:200, Dako, Stockholm, Sweden) or Iba1 (rabbit anti-rat, 1:200, Wako, Richmond, VA), then rinsed in PBS, incubated for 60 min with an appropriate fluorophore-conjugated secondary antibody (Alexa Fluor 594 donkey-anti-rabbit, 1:150 and Alexa Fluor 488, goat anti-rabbit, 1:300, both from Invitrogen), diluted in PBS and 0.3% Triton X-100, then rinsed in PBS and mounted in PBS-glycerol (1:3). Sections processed for immunohistochemistry were examined and captured in a Leica DM RBE microscope system (Leica).

### Astrocyte and microglia cultures

Primary astrocytes and microglia were isolated from adult DA and PVG brains of 10-week-old rats perfused via the ascending aorta with ice-cold PBS containing heparin. After removing the meninges and the cerebellum, the brains were homogenized in enzymatic solution (116 mM NaCl, 5.4 mM KCl, 26 mM NaHCO_3_, 1 mM NaH_2_PO_4_, 1.5 mM CaCl_2_, 1 mM MgSO_4_, 0.5 mM EDTA, 25 mM glucose, 1 mM cysteine and 20 u/ml papain – all from Sigma) using a micro-scissor. The homogenate was incubated for 60 min with gentle stirring at 37°C, 5% CO_2_. Next, the digested tissue was transferred to a 50 ml conical tube. The enzymatic reaction was stopped by adding HBSS (Invitrogen, Sweden) with 20% FCS. The homogenate was spun down at 200 *g* for 7 min and the pellet resuspended in 2 ml of 0.5 mg/ml DNaseI (Roche, Sweden) in HBSS, then filtered through a 40 μm strainer (Becton Dickinson, Sweden) and transferred to 20 ml of 20% stock isotonic Percoll in HBSS. Another 20 ml pure HBSS was carefully added on top to create a Percoll gradient; the samples were then gently centrifuged at 200 *g* for 20 min. The resulting pellet was washed once in HBSS and the cells resuspended in DMEM/F12 complete medium, supplemented with 10% heat-inactivated FCS, penicillin-streptomycin (100 u/ml, 100 μg/ml), 2 mM L-glutamine (Life Technologies) and 30% M-CSF conditioned L929 cell line supernatant. The cells were then plated in 75cc tissue flasks (Sarstedt, Nümbrecht, Germany) coated with poly-l-lysine (Sigma) and incubated at 37°C and 5% CO_2_ in a humidified incubator. The medium was changed twice weekly until the cells became confluent (approximately 14 days). When full confluence of the cell layer was reached, the mixed glial cells were harvested using pre-warmed trypsin (Gibco).

To separate microglia and astrocytes from each other in the mixed glial cell culture, magnetic separation was used according to the manufacturer’s instructions. In brief, the mixed cell suspension was centrifuged at 300*g* for 10 min. The cell pellet was resuspended in MACS buffer (Miltenyi Biotec GmbH, Germany) and stained with mouse anti-rat CD11b-PE (BD Biosciences, Sweden) for 10 min. Then, the cells were washed and resuspended in MACS buffer followed by AutoMACS magnetic separation using anti-PE MicroBeads (Miltenyi). The resulting microglia (CD11+ cells) and remaining cells (astrocytes) were seeded in 24-well plates (4 × 10^5^ cells/well and 2 × 10^5^ cells/well, respectively).

The cells (microglia and astrocytes) were then left unstimulated (only DMEM/F12 complete medium, supplemented with 10% heat-inactivated FCS, penicillin-streptomycin 100 u/ml, 100 μg/ml), or stimulated with recombinant rat TNF-α (R&D Systems, Minneapolis, MN) at 20 ng/ml (in the same medium) for 24 h after which the cells were lysed for RNA extraction and subsequent RT-PCR expressional analysis. The purities of microglia and astrocytes were checked with flow cytometry as described above using PE-labeled mouse anti-rat CD11b and FITC labeled mouse anti-rat GFAP antibodies (both from BD Pharmingen, Franklin Lakes, NJ). The purities of microglia and astrocytes were above 95% (data not shown).

### Statistical analysis

Partek uses ANOVA to test for expression differences with *P* < 0.01 chosen as the cut-off. In the studies of motor neuron survival, significance levels between two strains were calculated using the unpaired t-test (GraphPad Prism 5.0; San Diego, CA). In RT-PCR studies of multiple groups, one-way ANOVA with the Bonferroni *post hoc* test was used (GraphPad) to measure differences in mRNA expression as well as in the microarray expression studies. In the sorted T-cell studies, the unpaired t-test was used. In general *P* < 0.05 was considered statistically significant.

## Results

### Clec4a3 is differentially regulated following ventral root avulsion

**Figure 1:**
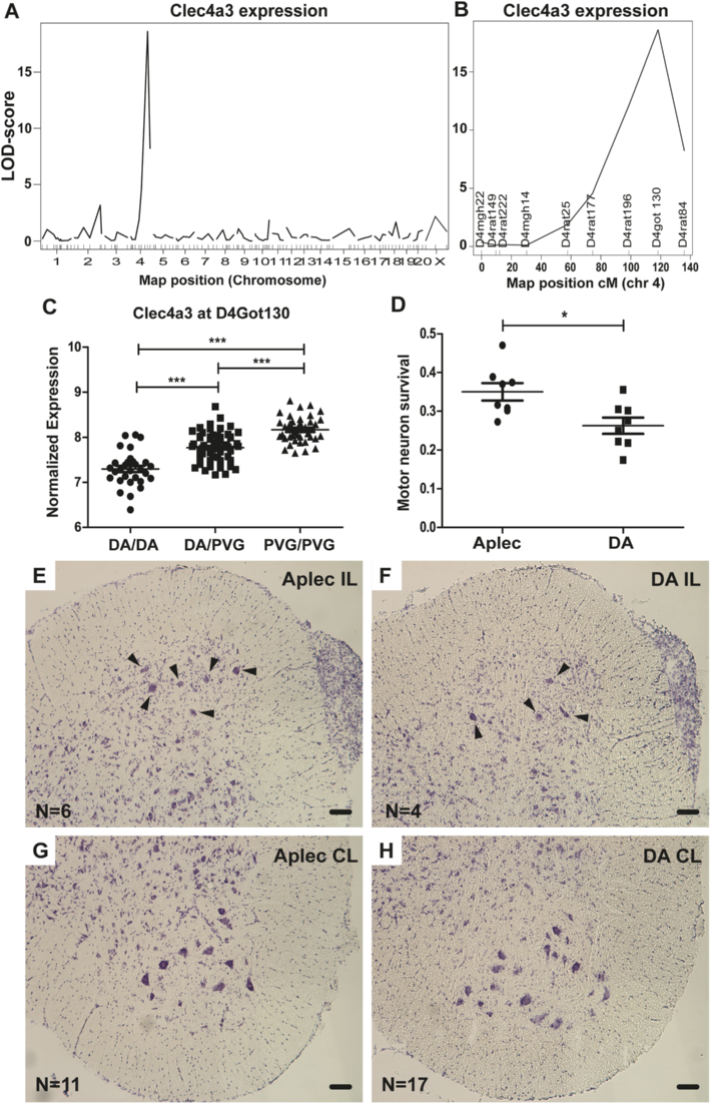
**Clec4a3 is *cis*-regulated after nerve root injury and the Aplec complex confers neuroprotection.** The inbred DA and PVG rat strains display considerable, genetically determined, differences in spinal cord motor neuron survival following VRA. Global expressional profiling of lesioned spinal cord ventral horn tissue from an F2 (DAxPVG) intercross reveals several strong eQTLs. One of the strongest eQTLs among more than 27,000 transcripts is Clec4a3, a C-type lectin with hitherto unknown function in the CNS. Clec4a3 was *cis*-regulated from chromosome 4 (**A**) from marker D4Got130 (**B**). All F2 animals were genotyped over the whole genome and the PVG genotype in marker D4Got130 was found to lead to higher expression of Clec4a3 (**C**), with highest expression in PVG homozygotes; the heterozygotes displayed an intermediate phenotype. The Aplec congenic strain, which has a small fragment from PVG onto DA background containing six characterized C-type lectin genes including Clec4a3, displays significantly improved motor neuron survival compared to DA at 21 days after VRA (**D**). A representative section from the L4 segment shows motor neurons in the ventral horn on the contralateral (CL) and ipsilateral (IL) sides of the cord (axotomized cells are indicated by arrows) in one Aplec (**E** and **G**) and one DA (**F** and **H**) rat. In A the x-axis marks each chromosome and in B it is the genetic distance on chromosome 4 measured in centimorgans (cM); the y-axis shows the logarithm of odds (LOD)-score, where LOD > 3.1 corresponds to a genome wide *P* < 0.001, and the current LOD-score of around 20 to *P* = 0. The scale bar in the micrographs corresponds to 80 μm. Aplec: antigen-presenting lectin-like receptor gene complex; CL: contralateral; CNS: central nervous system; eQTL: expression quantitative trait loci; IL: ipsilateral; LOD: logarithm of odds; VRA: ventral root avulsion.

The inbred rat strains DA and PVG differ substantially in degree of nerve cell death following VRA, with almost a twofold increased survival in the PVG strain compared to DA [26,38]. Since early reactions following injury likely affect downstream events, such as nerve cell death, we set up an F2 (DAxPVG) intercross and performed global transcriptional profiling of injured spinal cords combined with whole-genome linkage analysis to define eQTLs. Multiple eQTLs were identified and they will be presented separately (unpublished results). Interestingly, one of the strongest regulated eQTLs in the whole data set was Clec4a3, also called Dcir3, a C-type lectin receptor (Figure 1A-B), which does not have a defined role in the CNS (Table 2). Expression of Clec4a3 was higher in animals carrying PVG alleles at the peak marker D4Got130 (Figure 1C). MHCII-related genes [39] and glutathione-related genes [26] were also found to be highly differentially regulated between DA and PVG rats and have been reported previously.

**Table 2:** The 20 strongest regulated eQTLs in the F2(DAxPVG) intercross

**Transcript ID**	**Gene symbol**	**Gene name**	**Locus**	**Chr**	**cM**	**LOD score**	***P*Value**
10926967	LOC501110	Similar to glutathione S-transferase A1 (GTH1) (HA subunit 1) (GST-epsilon) (GSTA1-1)	D9Rat130	9	57	65.6988845987	0
10824140	Msr2_predicted	Macrophage scavenger receptor 2 (predicted)	D2Rat44	2	15	55.6575859002	0
10877130	Ltb4dh	Leukotriene B4 12-hydroxydehydrogenase	D5Rat77	5	36	39.6332668113	0
10908861	Ldha	Lactate dehydrogenase A	D1Rat265	1	2	38.7544785249	0
10802013	Cd74	CD74 antigen (invariant polypeptide of major histocompatibility complex, class II antigen-associated)	D10Mgh25	10	62	37.3794915401	0
10711664	Acadsb	Acyl-Coenzyme A dehydrogenase, short/branched chain	D1Arb21	1	7	35.2231095445	0
10828344	RT1-Da	RT1 class II, locus Da	D10Mgh25	10	62	32.6411125813	0
10831567	RT1-Bb	RT1 class II, locus Bb	D10Mgh25	10	62	31.6910114967	0
10784621	Ephx2	Epoxide hydrolase 2, cytoplasmic	D15Rat123	15	87	30.8274874187	0
10714907	Ifit1	Interferon-induced protein with tetratricopeptide repeats 1	D1Rat301	1	9	27.9442878525	0
10829888	Pbld	Phenazine biosynthesis-like protein domain containing	D20Rat7	20	107	25.4372427332	0
10725724	Eif3c	Eukaryotic translation initiation factor 3, subunit C	D1Arb21	1	7	25.4264147505	0
10812515	Arsb	Arylsulfatase B	D2Rat3	2	12	23.7304626898	0
10725778	Nupr1	Nuclear protein 1	D1Arb21	1	7	23.4051659436	0
10866041	Klrk1	Killer cell lectin-like receptor subfamily K, member 1	D4Got130	4	31	21.7980872017	0
10825931	MGC108896	Similar to glutathione transferase GSTM7-7	D2Rat44	2	15	21.7509939262	0
10725846	Ccdc95	Coiled-coil domain containing 95	D1Arb21	1	7	21.0341839479	0
10856673	Slc4a5	Solute carrier family 4, sodium bicarbonate cotransporter, member 5	D4Rat177	4	29	20.8720767896	0
10858559	Clec4a3	C-type lectin domain family 4, member a3 (Dcir3)	D4Got130	4	31	20.3859516269	0
10916458	RGD1309108_predicted	Similar to hypothetical protein FLJ23554 (predicted)	D8Rat41	8	50	20.0893368764	0

eQTL: expression quantitative trait loci.

### C-type lectins regulate motor neuron survival following VRA

Given a strong genetic influence on the expression of Clec4a3 and co-regulation of the expression of multiple C-type lectins with complement proteins such as C1q (unpublished results), which in turn is associated with degeneration of motor neurons following VRA [40], we set out to explore if genetic differences in C-type lectins also affect motor neuron survival. DA and congenic Aplec rats (containing a small PVG insert on a DA background with seven functional genes, all C-type lectins) were operated with unilateral VRA and allowed to survive for 21 days after which motor neuron survival on the injured side was assessed. DA rats had an average motor neuron survival of 26%, in line with previous studies [26,37], whereas Aplec rats displayed a significantly increased motor neuron survival, with an average survival of 35% (*P* < 0.05; Figure 1D-H). The PVG strain has previously been demonstrated to have a motor neuron survival of 40% [26].

### Expressional profiling of DA and Aplec rats reveals differences in inflammatory pathways and T-cell activation

To explore early post-injury processes that differ between DA and Aplec rats, we performed a microarray scan of the gene expression profiles in L4 spinal cord segments at 5 days after VRA, that is, the same time point used in the study of the F2 rats. Analysis of the global expressional profiling data showed 229 transcripts that differed between Aplec and DA rats at *P* < 0.01. All these transcripts were found also to differ between naïve DA and Aplec rats, indicating that they were part both of a strain and injury response (Additional file 1: Table S1). Clec4a3, one of the seven Aplec genes, was the transcript differing most between operated DA and Aplec rats and the second most differing transcript between naïve and operated Aplec rats. The identified genes were clustered for pathway enrichment using the DAVID functional annotation tool [31,32]. As expected, many clusters involved metabolic, neurodevelopmental and inflammatory processes. Also not surprisingly, the cluster with the highest enrichment score contained several of the genes in the Aplec complex, and was enriched for carbohydrate binding (*P* = 0.016) and C-type lectins (*P* = 0.022). The cluster included the genes Clec4a3 (Dcir3), Clec4a2 (Dcir2) and Clecsf6 (Dcir1), which are all included in the Aplec region, but also Cd69 (Clec2c) (Table 3). Apart from being a C-type lectin, CD69 is also a marker of activated T cells [12].

**Table 3:** The most significantly enriched gene cluster out of the differentially regulated genes

**Transcript ID**	**Gene symbol**	**Gene name**
10858573	Clecsf6, Dcir1	C-type (calcium dependent, carbohydrate recognition domain) lectin, superfamily member 6
10858559	Clec4a3, Dcir3	C-type lectin domain family 4, member a3
10865993	Clec2c, Cd69	Cd69 molecule
10880738	Epha8	Eph receptor A8
10858566	Clec4a2, Dcir2	Dendritic cell inhibitory receptor 2
10773298	Mrfap1	Mannosidase 2, alpha B2
10935047	Ngfrap1	Nerve growth factor receptor (TNFRSF16) associated protein 1
10847156	Olr673	Olfactory receptor 673
10823970	Tlr2	Toll-like receptor 2
10772768	Tlr6	Toll-like receptor 6
10703618	Vom2r21	Vomeronasal 2 receptor, 17; vomeronasal 2 receptor, 20; vomeronasal 2 receptor, 18

**Figure 2:**
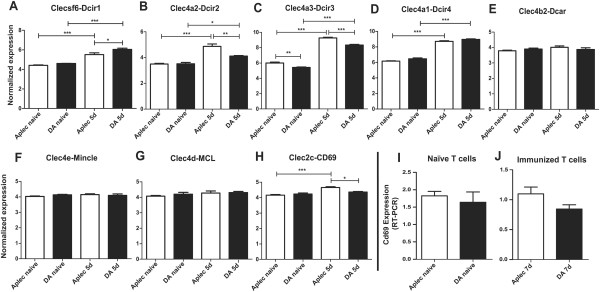
**Microarray studies of expression of the Aplec complex genes and Cd69 following VRA and the specific characterization of Cd69 expression in T cells.** Microarray expressional profiling of spinal cords from naïve and operated DA and Aplec rats was performed. A detailed study of all genes in the Aplec segment was performed, as well as of Cd69, a C-type lectin commonly used as a marker of T-cell activation, as bioinformatical enrichment analysis had revealed differential regulation of Cd69 between the strains. Clecsf6/Dcir1, Clec4a2/Dcir2, Clec4a3/Dcir3 and Clec4a1/Dcir4 were upregulated following injury in both strains (**A**-**D**). Expression of Clec4a3/Dcir3 and Clec4a2/Dcir2 was higher in Aplec rats and Clecsf6/Dcir1 in DA rats following VRA (**A**-**C**). Clec4b2/Dcar1, Clec4d/Mcl and Clec4e/Mincle were not regulated by either injury or strain (**E**-**G**). Cd69, which is not a part of the Aplec complex, was upregulated only in Aplec rats following injury (**H**). To decide whether the differential upregulation of Cd69 was caused by inherent T-cell differences, or rather by an increased T-cell influx, characterization of Cd69 expression was performed in naïve and stimulated T cells from DA and Aplec rats. This revealed no significant strain differences in either naïve (**I**) or stimulated (**J**) T cells, suggesting the increased expression in the Aplec spinal cords is derived from an increased number of T cells. VRA: ventral root avulsion.

Of the seven genes in the Aplec complex Clec4a1 (Dcir4), Clec4a2, Clec4a3 and Clecsf6 were upregulated following injury in both strains, but with a higher expression of Clec4a3 and Clec4a2 in Aplec rats and of Clecsf6 in DA rats. Clec4b2 (Dcar1), Clec4d (Mcl) and Clec4e (Mincle) were not significantly regulated, either by VRA or by strain (Figure 2A-G). Cd69, which does not physically locate to the Aplec complex, was upregulated by VRA only in the Aplec strain (Figure 2H). Replication with RT-PCR was performed on L3 segments from the same animals as used in the microarray study to confirm the expression of most of the above mentioned genes, except for Mincle and Mcl, which were lowly expressed and without differences between strains. The RT-PCR expression showed similar results as the microarray (Additional file 2: Figure S1).

### Increased Cd69 expression likely reflects an increased number of T cells

Increased Cd69 expression after VRA, seen in Aplec but not DA rats, indicated potential involvement of T cells in the injury response. As quantification of Cd69 expression cannot distinguish differences in cellular composition, that is, infiltration of cells, from a relative increase in expression across cells, we characterized T cells from both strains regarding Cd69 expression. MOG immunization was performed in DA and Aplec rats, and T cells were sorted from inguinal lymph nodes 7 days after immunization, before clinical EAE developed. T cells were also sorted from inguinal lymph nodes of naïve animals from both strains. Cd69 expression in the T cells was subsequently analyzed with RT-PCR. There was no difference in Cd69 expression between Aplec and DA T cells, neither in the naïve nor the immunized state (Figure 2I,J). Cd4 and Cd8 expression was also identical between strains (data not shown). This suggests that the increased Cd69 expression in the spinal cord of Aplec rats compared to DA rats comes from an increase in the number of T cells, rather than a differential regulation of Cd69 expression *per se*.

### T-cell infiltration differs between Aplec, PVG and DA rats following VRA

**Figure 3:**
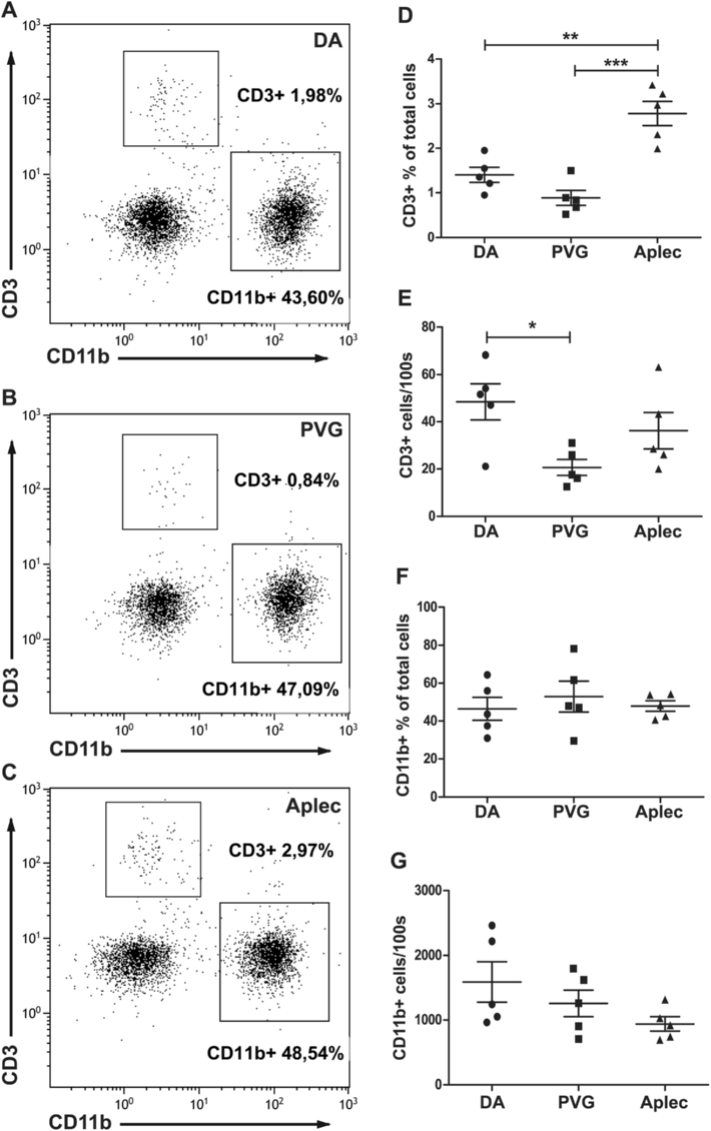
**Increased motor neuron survival in the Aplec strain is associated with more prominent T-cell infiltration.** Microarray expressional profiling in Aplec rats suggested involvement of T cells. To confirm this, ipsilateral L3-5 spinal cord segments from DA, PVG and Aplec rats were analyzed with flow cytometry 5 days after VRA. The relative proportion of CD3+ cells was significantly increased in Aplec rats compared to both DA and PVG rats (**A**-**D**). **A**-**C** show representative flow cytometry plots from one animal in each group. To approximate the absolute number of CD3+ cells in the tissue, the total number of cells acquired/100 seconds was measured in each animal, demonstrating more CD3+ cells in DA rats compared to PVG rats, but there were no significant differences between DA and Aplec rats suggesting a similar absolute number of T cells in the strains, but with a relatively larger proportion of T cells in Aplec rats. Also CD11b+ cells were analyzed, revealing around 50% of the cells in the tissue to be CD11b+ with no strain differences (**A**-**C** and **F**), although there was an insignificant trend for DA rats to have a higher total number of CD11b+ cells (**G**). Aplec: antigen-presenting lectin-like receptor gene complex; VRA: ventral root avulsion.

To further confirm the strain differences regarding T cells, DA, PVG and Aplec rats were subjected to VRA and spinal cord tissue was collected at 5 days after injury for flow cytometry. PVG rats were included for comparative purposes as this phenotype has not previously been determined in this strain. However, in a previous study infiltration of CD3+ cells was increased in DA compared to PVG as assessed by immunohistochemistry [37]. CD3 was the only T-cell marker used as only a small number of T cells was expected: a previous FACS study reported between 0 and 2% CD3+ cells in the spinal cord (of Sprague–Dawley rats) following injury [41]. In DA rats, on average 1.4% of the cells in the injured spinal cord were CD3+, in PVG rats 0.8% and Aplec rats 2.7% (Figure 3A-D), which demonstrates that the C-type lectin genes affect not only motor neuron survival but also the degree of T-cell infiltration into the cord. The above flow-cytometry analysis results are based on relative numbers of CD3+ cells. In order to obtain a more absolute measure of the number of CD3+ cells, we quantified CD3+ cells per acquisition time unit (see Materials and methods), which showed more CD3+ cells in DA rats compared to PVG rats, but there was no significant difference between DA and Aplec rats (Figure 3E). The difference between the analysis methods can likely be explained by the combined effect from a proportionally larger T-cell infiltration in Aplec rats and a slightly higher total cellular yield in the DA rats, caused by a greater infiltration/expansion of other, not labeled, cell populations.

CD11b+ cells, which mainly reflect activated microglia but to a lesser degree also other cell types such as monocytes and macrophages, were also quantified. Around 50% of all cells in the spinal cord were CD11b+ with no differences between Aplec and DA rats (Figure 3F). The approximation of the absolute numbers of CD11b+ cells between the strains did not result in any strain differences, although there was an insignificant trend for more CD11b+ cells in DA rats (Figure 3G).

### Glial activation is not affected by the Aplec complex

**Figure 4:**
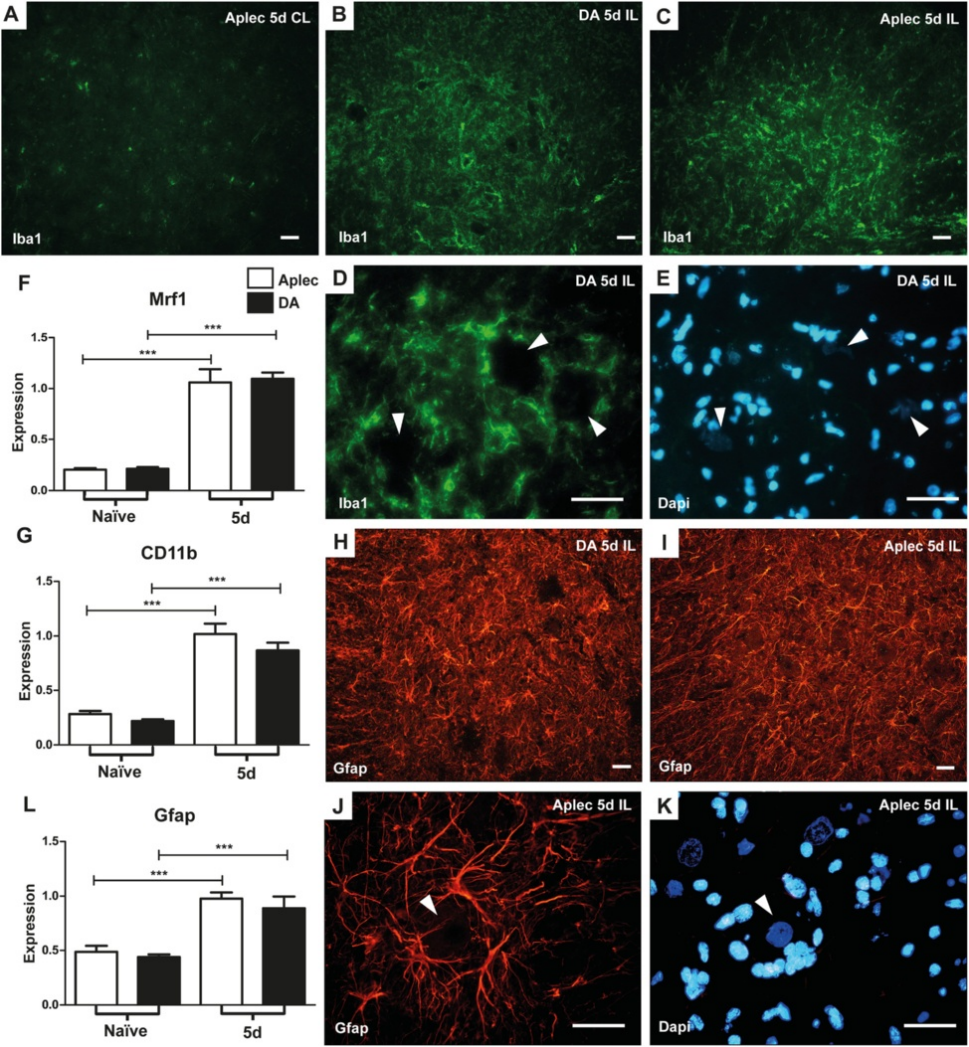
**Glial activation is not affected by the Aplec complex.** Glial activation is an important feature of the VRA injury response, and potential differences between DA and Aplec rats following VRA were analyzed. Assessment of microglia activation by RT-PCR quantification of CD11b and Mrf-1 expression (**F**,**G**) and immunolabeling using Iba1 was performed (**A**-**E**), illustrating largely identical patterns between DA and Aplec rats, which suggests a similar degree of microglia activation. Microglia can be seen surrounding the motor neurons, here indicated with white arrows, which are also easily distinguished by their larger size and more diffusely stained nucleus (**D**,**E**). Also astrocyte activation, assessed by RT-PCR quantification of Gfap expression (**L**) and immunolabeling (**H**-**K**) illustrated similar patterns between the strains. Astrocytes can be seen surrounding the motor neurons; one motor neuron is marked with a white arrow (**J**,**K**). Micrographs B, C, H and I show an overview of the ipsilateral (IL) ventral horn, whereas A shows the contralateral (CL), healthy side, with clearly less microglial activation. The scale bars correspond to 40 μm. Aplec: antigen-presenting lectin-like receptor gene complex; CL: contralateral; IL: ipsilateral; RT-PCR: real-time polymerase chain reaction; VRA: ventral root avulsion.

Since glial activation is a prominent feature following VRA [38] and likely involved in the complex reactions following injury, this was assessed in DA and Aplec rats following VRA. Both activation of microglia, assessed by quantifying CD11b and Mrf-1 expression (Figure 4F,G), and astrocytes, assessed by quantifying Gfap expression (Figure 4L), occur in the two strains, but without strain differences, suggesting that there are no major differences in the general glial activation pattern between Aplec and DA rats 5 days after VRA. To further analyze glial activation, immunolabeling using Gfap and Iba1 was performed, illustrating largely identical staining patterns between DA and Aplec rats, again suggesting a similar degree of general glial activation (Figure 4A-E, H-K).

### Aplec genes are expressed by cultured microglia, but not astrocytes

**Figure 5:**
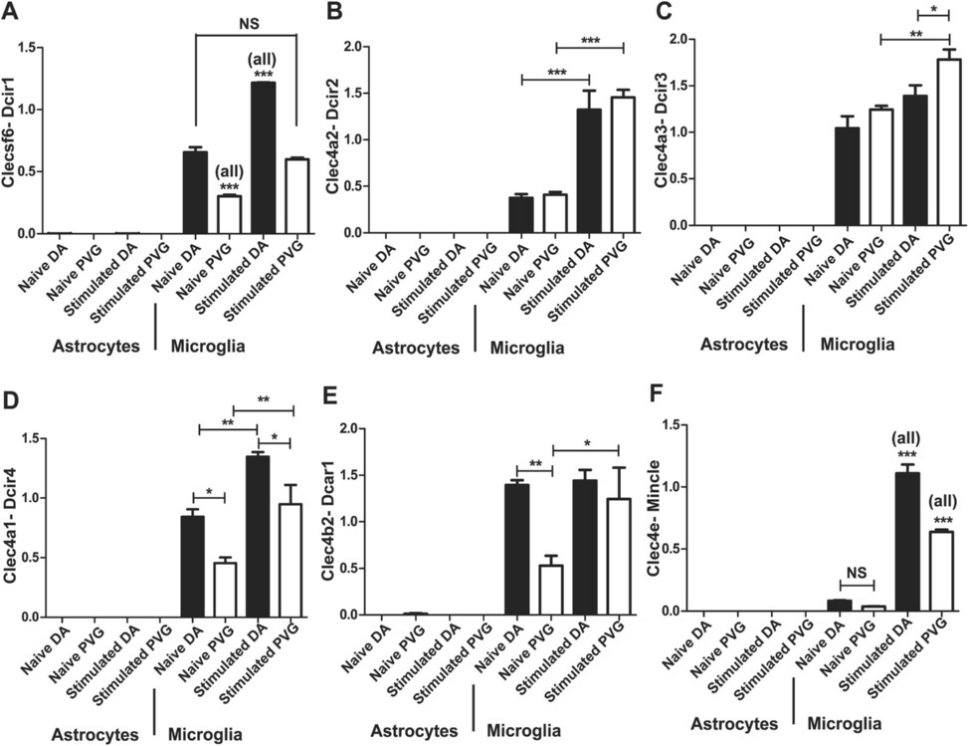
**Aplec genes are expressed by microglia, but not astrocytes, and several are upregulated following inflammatory stimulation.** C-type lectins are mostly known to be expressed by antigen-presenting cells (APCs). In order to determine the source of expression of the Aplec genes, primary astrocyte and microglia cultures were established from DA and PVG rats and exposed to stimulation with TNF-α. Expression of all the Aplec genes was more than 1000-fold higher in microglia compared to astrocytes, thus demonstrating that the C-type lectins here studied are expressed by microglia (**A**-**F**). Also, there was strain differences exemplified for instance with upregulation of Clec4a3/Dcir3 only in the PVG microglia (**C**), while Clecsf6/Dcir1, Clec4a1/Dcir4, Clec4b2/Dcar1 and Clec4e/Mincle were more highly expressed in microglia derived from DA rats (**A**, **D**-**F**). Mcl is not shown, but the expression pattern is similar to that of Mincle. APC: antigen-presenting cell; Aplec: antigen-presenting lectin-like receptor gene complex.

To study the source of expression of C-type lectins in the CNS, for which existing information is very limited and there is a scarcity of commercially available antibodies, expression of all Aplec genes was studied in naïve and TNF-stimulated astrocytes and microglia derived from DA and PVG rats, respectively (Figure 5A-F). In general, expression of all genes was barely measurable in astrocytes and more than 1000-fold higher in microglia. Also, TNF-α stimulation led to upregulation of Clecsf6, Clec4a1 and Clec4a2 in both strains (Figure 5A,B,D), but only Clec4a3 in PVG rats (Figure 5C). Thus, C-type lectins are expressed in microglia but not astrocytes and they are upregulated by inflammatory stimuli.

### Analysis of M1, M2 and DC markers

**Figure 6:**
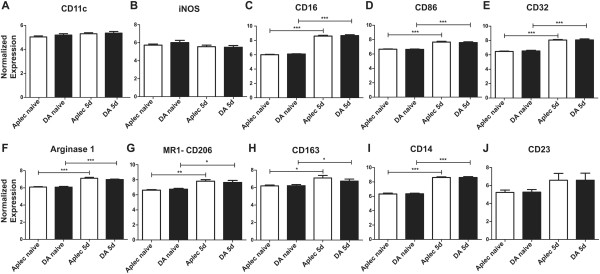
**Analysis of dendritic cell, M1 and M2 markers displays a similar pattern between DA and Aplec rats.** Global expressional profiling of spinal cords from naïve and operated DA and Aplec rats enabled a detailed study of CD11c, a marker commonly used for dendritic cells (DCs), as well as markers used to differentiate between M1 (pro-inflammatory) and M2 (anti-inflammatory) macrophages and microglia. CD11c expression was unaffected by strain or injury, arguing against large differences in DC activity (**A**). iNOS (inducible NO-synthase), CD16, CD86 and CD32 are typical M1 markers (**B**-**E**) whereas arginase 1, mannose receptor 1/CD206, CD163, CD14 and CD23 are typical M2 markers (**F**-**J**) and they all showed similar patterns without strain differences, where most were significantly upregulated in both DA and Aplec rats after injury. Aplec: antigen-presenting lectin-like receptor gene complex; DC: dendritic cell’ iNOS: inducible NO-synthase; MR1: mannose receptor 1.

Since CLECs are associated with DC levels of CD11c, expression was assessed in the microarray data to see if there were any major differences, which may reflect differences in the DC population. However, the CD11c levels were low, unaffected by injury, and similar between the strains arguing against large differences in DC populations (Figure 6A).

Also macrophages infiltrate the spinal cord after injury, and it was recently demonstrated that the polarization of macrophages towards either an M1 (expressing more iNOS, CD16, CD86 and CD32) or M2 (expressing more arginase 1, CD206/mannose receptor, CD163, CD14 and CD23) phenotype affects outcome after spinal cord injury in mouse [7]. The M1 and M2 phenotypes may also be relevant in microglia [42]. We therefore assessed expression levels of most known genes associated with either an M1 (Figure 6B-E) or M2 (Figure 6F-J) phenotype, and saw very similar patterns between the strains arguing against a strong differential polarization towards an M1 or M2 phenotype.

### Expression of neurotrophic factors is not affected by the Aplec complex

There are numerous growth factors that in different models have proven to exert beneficial effects on motor neuron survival and development such as neurotrophin-3 (Ntf3), brain-derived neurotrophic factor (Bdnf) and glial-derived neurotrophic factor (Gdnf) [43], pleiotrophin [44], as well as transforming growth factor β2 (Tgfb2) [45]. Expression levels of these neurotrophic factors did not differ between DA and Aplec rats in the microarray data set (Additional file 3: Figure S2A-H). In fact, the levels of most neurotrophic factors were largely unaffected by the nerve root injury, except for insulin growth factor 1 (Igf1) and transforming growth factor β1 (Tgfb1), which were both upregulated after injury, but without differences between strains (Additional file 3: Figure S2G,H). This suggests that C-type lectins do not primarily affect motor neuron survival by influencing the expression of neurotrophic factors.

### Further fine mapping of the Aplec region

**Figure 7:**
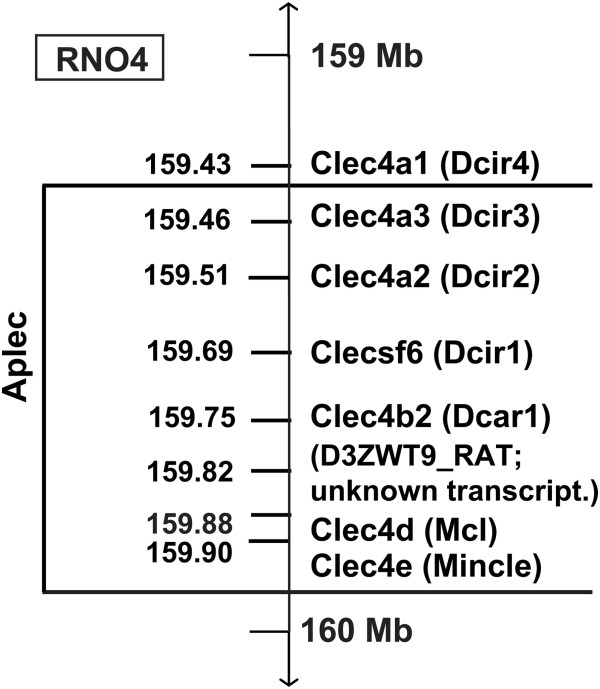
**Genetic architecture of the Aplec segment in detail.** The Aplec gene cluster is a very small segment located between 159.4 and 159.9 Mb of rat chromosome 4, RNO4. The Aplec congenic strain contains seven genes, all C-type lectins, out of which the gene D3ZWT9_Rat is poorly characterized, from PVG rats transferred onto DA background. Aplec: antigen-presenting lectin-like receptor gene complex.

The expression of Clec4a2 and Clec4a3 was higher in Aplec rats, while Clecsf6 was more expressed in DA rats, and Clec4a1 did not differ after injury, as demonstrated both by the microarray and RT-PCR. Dcar1, Mincle and Mcl were unaffected by VRA. According to the physical map of the Aplec complex, Clec4a1 is located outside the Aplec congenic region [19,22] (Figure 7), thus it does not differ between the Aplec congenic rats and DA rats, whereas Clec4a2, Clec4a3 and Clecsf6 are within the congenic region. As Clec4a3 was one of the strongest expression phenotypesin the F2(DAxPVG) intercross, the second most differentially regulated gene in microarray analysis between DA and Aplec rats and also differentially regulated in the microglia cultures, it constitutes the most interesting candidate gene, even though the positive contribution from Clec4a2 in Aplec rats, or the negative contribution from Clecsf6 in DA rats from cannot be ruled out.

## Discussion

We demonstrate that a genetic variation in a C-type lectin gene cluster (Aplec) occurring among inbred rat strains significantly affects medium-term neuronal survival and lymphocyte infiltration following a standardized proximal nerve root avulsion injury.

The function of C-type lectins has mostly been studied in the context of dendritic cell immunobiology and their function as antigen-presenting cells, which is relevant for shaping ensuing adaptive immune responses [46,47]. However, detailed knowledge about the roles of many C-type lectins is still lacking. Moreover, any possible involvement of CLECs in the reaction to traumatic CNS injuries is entirely unknown. There exist a few examples where CLECs have been implicated in CNS pathology, for example, Clec16a has been genetically associated with susceptibility to multiple sclerosis (MS) [48] and is upregulated in astrocytes after infectious stimuli [49], and Clec5a was recently demonstrated to have a disease regulatory effect in a mouse viral encephalitis model [50]. Since microglia display certain similarities to DCs and arguably constitute the main resident APCS in the CNS [2], it is not surprising that we found a much higher expression of C-type lectins on microglia compared to astrocytes. The strain differences in CLEC expression could suggest differential polarization of microglia in the Aplec and DA strains towards a phenotype more like either M1 (inflammatory) or M2 (anti-inflammatory). We therefore analyzed the expression of most known M1 or M2 markers; however, no such differences between strains were found. This does not exclude more subtle phenotypic variants not reflected by differences in the expression of traditional M1/M2 markers, or that the CLEC expression could be linked to another, not yet defined, microglia or macrophage subtype.

The CLECs are commonly associated with DCs, which in the healthy CNS constitute a very small population, even though they can increase in number in pathological conditions [51]. These cells may exert effects also in the context of neurotrauma. For example, injection of DCs immunized with whole spinal cord homogenate improved outcomes after spinal cord injury in mice [52] and also higher mammals [53]. However, we did not find differences in the expression of the DC marker CD11c, either in naïve animals or after injury. This finding argues against substantial differences in DC recruitment, but as for the lack of differences in the expression of M1/M2 microglia markers, it does not exclude more subtle differences in the DC activation pattern.

T cells are only rarely encountered in an intact CNS and infiltration is a tightly regulated process depending on recruitment/signaling events occurring in the tissue [54,55]. Thus, for example after a peripheral nerve injury, it is well established that T cells are recruited to the axotomized motor neuron pools [56], even if the exact molecular pathways leading to immune cell infiltration are not entirely understood. Notably, this infiltration may serve a functionally important purpose as the lack of immune cells in severe combined immunodeficient mice has been shown to aggravate the loss of axotomized cells after facial nerve axotomy [57,58]. This is also highly relevant for the finding here; namely, that a genetic variability in the Aplec cluster not only affects motor neuron survival, but as an earlier event, also infiltration of T cells to the cord. This also implies that these molecules are involved not only in regulating processes commonly associated with strong adaptive immune responses, such as occur in autoimmune disease models, but also more subtle immune activation patterns occurring in the CNS. Whether or not increased T-cell infiltration after VRA contributes to increased motor neuron survival cannot be concluded from the data presented here. However, accumulating experimental evidence suggests that T cells under some circumstances can mediate neuroprotective effects [10], including chronic models of neurodegeneration [11]. Also, in the VRA model using the DA strain, concomitant experimental autoimmune neuroinflammation has been shown to increase the survival of axotomized cells, which was also associated with the infiltration of large numbers of lymphocytes [59]. Interestingly, non-encephalitogenic bystander recruited T and natural killer (NK) cells were found to express several neurotrophic factors [59]. We here examined expression of several neurotrophic factors, including Ntf3, Bdnf and Gdnf, in the microarray data set without finding differences in the expression between Aplec and DA rats. There are also other proposed mechanisms by which T cells may confer neuroprotection, for example that T cells could deplete autoreactive/harmful microglia, as NK cells have been demonstrated to do [60]. However, the lack of differences between DA and Aplec rats regarding both the expression of neurotrophic factors and general markers of microglia activation does not provide support for these two proposed mechanisms, at least not in the setting studied here. Further potential mechanisms could be that T cells alter the microglia phenotype towards a more protective M2 phenotype [61] or to a more DC-like profile [11]. The opposite scenario is also possible, namely, that microglia instead affect the T cells [62], akin to how DCs can modulate T-cell activation [14,63]. We favor this view in the model studied here, since microglia activation starts well before T-cell infiltration following nerve injury [41]. On the other hand, it is important to acknowledge that the detrimental effects of T cells for nerve cells is also well established in many infectious and neuro-inflammatory conditions, such as EAE [64], and that further research is needed to define under what conditions T-cell responses may exert positive or negative effects on nerve cells. It should also be noted that we have here studied medium-term nerve cell survival and that differences between strains may level off in the long-term perspective.

Thus, our results provide strong support for the notion that the Aplec complex is of importance for communication between CNS-resident APCs, that is, microglia, and T cells. Interestingly, the Aplec strain displayed more infiltrating T cells than both the PVG strain, from which the Aplec complex derives, and the parental DA strain, which likely is a result of epistasis, a phenomenon not rarely seen in congenic rat strains[65,66]. Epistasis implies that there is a gene-gene interaction between the genes in the congenic fragment and genes residing in the background genome where the fragment is integrated. Due to the limited information available on binding partners for specific C-type lectins it is difficult to speculate on candidates for this effect, especially since this may not only depend on differences in expression but alsoon genetic variations affecting protein structure. Nevertheless, this is important to bear in mind when interpreting the large amount of seemingly disparate results concerning the effect of T cells and microglia in different experimental models, since the genetic set-up of the experimental animal will affect multiple cellular processes, which may be further amplified when studying complex *in vivo* reactions involving multiple cell types.

The CLEC family is complex; there are 25 rat paralogs to Clec4a3, out of which Clecsf6 (Dcir1), Clec4a2 (Dcir2), Clec4b2 (Dcar) and Clec4a1 (Dcir4) are clearly the most similar; all are located in the Aplec cluster and display around 50% sequence homology both at cDNA and protein level [25] with each other. Also, the above mentioned four CLECs are orthologs to human Clec4a (Dcir), which has been shown to be expressed mostly on DCs and assigned a role for modulating and shaping T-cell and general immune responses [63,67]. The mouse ortholog to Dcir has been functionally studied using Dcir^−/−^ mice, which displayed increased susceptibility to various autoimmune conditions characterized by excessive expansion of the DC pool [68]. Our findings are thus concordant with previous knowledge regarding CLECs, and especially Dcir and its rat homologs, suggesting that the genes act through APCs, in our case microglia, to regulate immune, and specifically T-cell responses. Due to the complex architecture of the gene family in both rats and mice, it is difficult to dissect the role of one gene from another; however, the eQTL mapping data from the F2(DAxPVG) intercross identifies Clec4a3 as a very interesting candidate for further studies. This could be done with extensive breeding to create sub-congenic strains, even if this may be very time consuming, or the creation of transgenic animals, which is now also possible for rats.

## Conclusions

We here demonstrate that a genetic variation occurring between inbred DA and PVG rats in the Aplec cluster, which encodes six characterized C-type lectin genes, significantly affects survival of axotomized motor neurons and T-cell infiltration after nerve root injury. Through extensive global expression profiling in an intercross between DA and PVG rats, one of the Aplec genes, Clec4a3 (Dcir3), was identified as one of the strongest *cis*-regulated eQTLs, thus, identifying it as a strong candidate to mediate the congenic phenotype. The function of Clec4a3 is still poorly studied, but in a small study it was found to have immunoregulatory functions in macrophages *in vitro*[69]. The finding of expression of the relevant C-type lectin transcripts in microglia suggests that they play an important role for recruiting/priming T cells. These results provide strong support for the notion that CLECs are important not only for shaping adaptive antigen-restricted immune responses occurring at a systemic level, but also localized inflammatory reactions occurring after nerve root injury and that this affects medium-term nerve cell survival. A more detailed characterization over time of the cellular and molecular processes that occur after VRA and how this is affected by the Aplec region is needed to provide a better understanding of underlying mechanisms. Also, further studies aimed at defining the ligands binding to C-type lectins and clarification of how signaling through C-type lectins prime APCs and shapes subsequent T-cell responses are warranted to clarify their role in basic neuroimmune interactions occurring both in primarily autoimmune neuroinflammation and conditions with inflammatory components, such as traumatic nerve injuries.

## Abbreviations

APC: Antigen-presenting cell; Aplec: Antigen-presenting lectin-like receptor gene complex; Bdnf: Brain-derived neurotrophic factor; BSA: Bovine serum albumin; CL: Contralateral; CLEC: C-type lectin; CNS: Central nervous system; DC: Dendritic cell; Dcir: Dendritic cell immunoreceptor; EAE: Experimental allergic encephalomyelitis; eQTL: Expression quantitative trait loci; FCS: Fetal calf serum; Gdnf: Glial-derived neurotrophic factor; Hbss: Hank’s balanced salt solution; IFA: Incomplete Freund’s adjuvant; Igf1: Insulin growth factor 1; IL: Ipsilateral; IVT: *in vitro* transcription; LOD: Logarithm of odds; MHC: Major histocompatibility complex; MIAME: Minimal information about a microarray experiment; MOG: Myelin oligodendrocyte glycoprotein; NK: Natural killer; Ntf3: Neurotrophin-3; rMOG: Recombinant myelin oligodendrocyte glycoprotein; PBS: Phosphate-buffered saline; RT-PCR: Real-time polymerase chain reaction; Tgfb1: Transforming growth factor β1; Tgfb2: Transforming growth factor β2; TNF: Tumor necrosis factor; tris-EDTA: Tris(hydroxymethyl)aminomethane (Tris)- Ethylenediaminetetraacetic acid (EDTA); VRA: Ventral root avulsion.

## Competing interests

All authors declare that they have no competing interests.

## Authors’ contributions

RL designed the study, performed or participated in all the experiments, analyzed the data and prepared the manuscript. SA performed the experiments, especially the animal experiments and the expression analysis, and analyzed the data. RP performed the flow-cytometry experiments and analyzed the data. FAN performed the experiments, especially the animal and flow-cytometry experiments. XZ performed the cell culture experiments. MS performed the animal experiments. CD analyzed the data from the cell culture experiments. SF performed the MOG immunization and T-cell sorting. MD performed the experiments and assisted in the planning. FP performed the experiments, provided funding, planned the experiments and prepared the manuscript. All authors read and approved the final manuscript.

## Supplementary Material

Additional file 1: Table S1 List of differentially regulated genes (*P* < 0.01) between DA and Aplec rats, and between naïve and injured Aplec rats. The table shows the genes differentially expressed between DA and Aplec rats following VRA. Click here for file

Additional file 2: Figure S1 RT-PCR confirmation of the expression of the Aplec genes and Cd69. To confirm the results from the microarray expressional profiling of the DA and Aplec spinal cords, RT-PCR analysis of expression in the L3 segments from naïve and VRA operated animals from both strains was performed. The RT-PCR quantification confirmed the microarray results; Clecsf6 (Dcir1), Clec4a2 (Dcir2), Clec4a3 (Dcir3) and Clec4a1(Dcir4), were all upregulated following injury in both strains, but with higher expression of Clec4a2 and Clec4a3 in Aplec and of Clecsf6 in DA and no strain differences in Clec4a1 **(A-D)**. Dcar showed no injury or strain variation **(E)**. Cd69 was significantly upregulated only in the Aplec strain **(F)**. Click here for file

Additional file 3: Figure S2 Expression of neurotrophic factors in DA and Aplec rats. Expression of the most acknowledged factors with neurotrophic effects was assessed in the microarray expressional profiling data set. Expression of **(A)** neurotrophin-3 (Ntf3), **(B)** brain-derived neurotrophic factor (Bdnf), **(C)** glial-derived neurotrophic factor (Gdnf), **(D)** nerve growth factor (Ngf), **(E)** pleiotrophin and **(F)** transforming growth factor β2 (Tgfb2), was regulated by neither injury nor strain. In contrast, the expression of **(G)** transforming growth factor β1 (Tgfb1) and **(H)** insulin growth factor 1 (Igf1) was upregulated in both strains, but without differences. Click here for file
