# Supplementary material for: Genetic variability in the rat Aplec C-type lectin gene cluster regulates lymphocyte trafficking and motor neuron survival after traumatic nerve root injury
Source: J Neuroinflammation. 2013 May 8;10:60. doi: 10.1186/1742-2094-10-60 (PMC3661385; doi:10.1186/1742-2094-10-60)
Supplement: Additional file 1: Table S1 — List of differentially regulated genes (P < 0.01) between DA and Aplec rats, and between naïve and injured Aplec rats. The table shows the genes differentially expressed between DA and Aplec rats following VRA. [file 1742-2094-10-60-S1.doc]

## Additional table 1. List of differentially regulated genes (p<0.01) between VRA operated DA and Aplec, and between injured and naïve Aplec.

|  |  | **Aplec Operated vs DA Operated** | |  | **Aplec: Naive vs Operated** | |  |
| --- | --- | --- | --- | --- | --- | --- | --- |
| **Transcript ID** | **Gene Symbol** | **p-value** | **Ratio** | **Fold-Change** | **p-value** | **Ratio** | **Fold-Change** |
| 10930569 | --- | 1.55E-06 | 0.492624 | -2.02995 | 1.58E-08 | 2.65747 | 2.65747 |
| **10858559** | **Clec4a3** | **2.65E-06** | **1.93879** | **1.93879** | **1.64E-13** | **0.104531** | **-9.56657** |
| 10791394 | Ddx60 | 9.63E-06 | 0.643949 | -1.55292 | 1.94E-06 | 0.617806 | -1.61863 |
| 10771881 | RGD1561381 | 2.34E-05 | 0.712212 | -1.40408 | 1.16E-07 | 1.66642 | 1.66642 |
| 10713608 | --- | 2.96E-05 | 1.74922 | 1.74922 | 5.27E-06 | 0.536546 | -1.86377 |
| 10787889 | Ddx60 | 3.71E-05 | 0.679346 | -1.472 | 1.56E-06 | 0.612762 | -1.63195 |
| 10910764 | --- | 4.07E-05 | 1.75619 | 1.75619 | 1.82E-05 | 0.561552 | -1.78078 |
| 10767266 | --- | 4.09E-05 | 0.703336 | -1.4218 | 3.85E-04 | 1.30193 | 1.30193 |
| 10847817 | Elf5 | 5.49E-05 | 1.28771 | 1.28771 | 9.79E-03 | 0.881844 | -1.13399 |
| 10723898 | --- | 6.43E-05 | 1.68321 | 1.68321 | 8.04E-05 | 0.616975 | -1.62081 |
| 10721698 | --- | 7.68E-05 | 2.85981 | 2.85981 | 1.07E-06 | 0.228021 | -4.38556 |
| 10804339 | --- | 8.18E-05 | 1.29008 | 1.29008 | 4.98E-04 | 0.819817 | -1.21978 |
| 10843357 | Tubb2c | 1.09E-04 | 0.805259 | -1.24184 | 3.82E-05 | 1.25652 | 1.25652 |
| 10803049 | --- | 1.33E-04 | 1.59356 | 1.59356 | 3.27E-05 | 0.600815 | -1.66441 |
| 10815622 | Aadac | 1.42E-04 | 1.14292 | 1.14292 | 2.96E-04 | 0.88955 | -1.12416 |
| 10701475 | --- | 1.78E-04 | 0.543792 | -1.83894 | 3.90E-05 | 1.96392 | 1.96392 |
| 10721099 | Tdrd12 | 1.86E-04 | 1.14836 | 1.14836 | 2.88E-07 | 0.787969 | -1.26909 |
| 10834604 | --- | 1.91E-04 | 2.10543 | 2.10543 | 1.40E-05 | 0.399938 | -2.50039 |
| 10812127 | Use1 | 2.21E-04 | 1.19039 | 1.19039 | 2.67E-06 | 0.776416 | -1.28797 |
| 10878961 | --- | 3.12E-04 | 2.0204 | 2.0204 | 1.19E-05 | 0.395575 | -2.52797 |
| 10791358 | Ddx60 | 3.64E-04 | 0.727384 | -1.37479 | 5.72E-07 | 0.572591 | -1.74645 |
| 10765040 | --- | 3.74E-04 | 1.96369 | 1.96369 | 1.08E-05 | 0.399791 | -2.5013 |
| 10702293 | --- | 4.52E-04 | 2.09196 | 2.09196 | 3.51E-03 | 0.583363 | -1.7142 |
| 10779099 | Ppp3cb | 4.66E-04 | 0.855185 | -1.16934 | 2.15E-06 | 1.28621 | 1.28621 |
| 10758033 | --- | 4.67E-04 | 2.70385 | 2.70385 | 3.87E-05 | 0.292931 | -3.41377 |
| **10858566** | **Clec4a2** | **5.02E-04** | **1.60524** | **1.60524** | **3.32E-07** | **0.40651** | **-2.45997** |
| 10709825 | --- | 6.07E-04 | 1.86458 | 1.86458 | 7.54E-05 | 0.476197 | -2.09997 |
| 10792302 | --- | 6.61E-04 | 0.797598 | -1.25376 | 3.03E-04 | 1.26451 | 1.26451 |
| 10802639 | Ccdc11 | 7.03E-04 | 1.16886 | 1.16886 | 1.10E-04 | 0.833678 | -1.1995 |
| 10715211 | Cyp2c11 | 7.30E-04 | 1.12031 | 1.12031 | 4.40E-06 | 0.834486 | -1.19834 |
| 10940466 | --- | 7.31E-04 | 0.911761 | -1.09678 | 2.74E-03 | 1.0767 | 1.0767 |
| 10700066 | --- | 7.45E-04 | 1.2701 | 1.2701 | 6.02E-05 | 0.741951 | -1.3478 |
| 10853755 | Ppia | 9.06E-04 | 0.851494 | -1.17441 | 4.92E-04 | 1.17827 | 1.17827 |
| 10700059 | --- | 1.18E-03 | 1.12252 | 1.12252 | 1.14E-04 | 0.866779 | -1.1537 |
| 10921680 | Cnpy3 | 1.19E-03 | 1.2136 | 1.2136 | 5.06E-08 | 0.621256 | -1.60964 |
| 10778137 | Nefh | 1.21E-03 | 0.835669 | -1.19665 | 2.37E-07 | 1.47788 | 1.47788 |
| 10793748 | LOC680692 | 1.29E-03 | 1.13829 | 1.13829 | 2.27E-12 | 0.503203 | -1.98727 |
| 10889568 | Dus4l | 1.36E-03 | 1.32503 | 1.32503 | 7.92E-03 | 0.812606 | -1.23061 |
| 10863676 | Egr4 | 1.41E-03 | 0.768693 | -1.30091 | 7.03E-05 | 1.41917 | 1.41917 |
| 10844223 | Ptges | 1.55E-03 | 0.745021 | -1.34224 | 1.07E-06 | 0.558466 | -1.79062 |
| 10912218 | Plscr1 | 1.61E-03 | 1.25123 | 1.25123 | 2.29E-08 | 0.543237 | -1.84082 |
| 10919266 | Ankrd34c | 1.64E-03 | 0.77231 | -1.29482 | 3.10E-05 | 1.46249 | 1.46249 |
| 10867950 | --- | 1.67E-03 | 1.27501 | 1.27501 | 5.54E-04 | 0.767818 | -1.30239 |
| 10805227 | --- | 1.79E-03 | 1.23628 | 1.23628 | 1.58E-04 | 0.765195 | -1.30686 |
| 10902910 | --- | 1.85E-03 | 0.816743 | -1.22437 | 9.04E-05 | 1.31256 | 1.31256 |
| 10852054 | --- | 1.86E-03 | 1.25135 | 1.25135 | 1.83E-04 | 0.755823 | -1.32306 |
| 10811732 | Sult5a1 | 1.94E-03 | 0.86573 | -1.15509 | 1.72E-03 | 1.14903 | 1.14903 |
| 10805225 | --- | 2.06E-03 | 1.34483 | 1.34483 | 4.51E-03 | 0.777551 | -1.28609 |
| 10877372 | Mup5 | 2.09E-03 | 0.728692 | -1.37232 | 3.68E-03 | 1.31983 | 1.31983 |
| 10836504 | Xirp2 | 2.18E-03 | 1.75155 | 1.75155 | 4.00E-06 | 0.355236 | -2.81503 |
| 10752959 | --- | 2.25E-03 | 0.841728 | -1.18803 | 4.26E-03 | 1.16106 | 1.16106 |
| 10842675 | --- | 2.26E-03 | 1.38443 | 1.38443 | 4.80E-04 | 0.687681 | -1.45416 |
| 10892173 | --- | 2.28E-03 | 0.754445 | -1.32548 | 1.47E-05 | 1.59168 | 1.59168 |
| 10761375 | Vkorc1l1 | 2.38E-03 | 0.910528 | -1.09826 | 2.95E-06 | 1.19713 | 1.19713 |
| 10849655 | Kcnip3 | 2.48E-03 | 0.725144 | -1.37904 | 1.66E-06 | 1.91908 | 1.91908 |
| 10821115 | --- | 2.50E-03 | 1.53655 | 1.53655 | 6.27E-03 | 0.70029 | -1.42798 |
| 10818304 | --- | 2.52E-03 | 1.28858 | 1.28858 | 2.92E-03 | 0.790094 | -1.26567 |
| 10932793 | --- | 2.53E-03 | 1.3138 | 1.3138 | 2.01E-07 | 0.515248 | -1.94081 |
| 10823970 | Tlr2 | 2.54E-03 | 0.803226 | -1.24498 | 1.37E-08 | 0.51677 | -1.9351 |
| 10715984 | RGD1305481 | 2.56E-03 | 1.24119 | 1.24119 | 2.51E-03 | 0.814163 | -1.22826 |
| 10806585 | Junb | 2.62E-03 | 1.28922 | 1.28922 | 1.45E-06 | 0.591262 | -1.6913 |
| 10920979 | Xirp1 | 2.63E-03 | 1.28107 | 1.28107 | 2.75E-04 | 0.733419 | -1.36348 |
| 10900628 | Hmha1 | 2.68E-03 | 1.27514 | 1.27514 | 7.04E-10 | 0.395619 | -2.52769 |
| 10702192 | --- | 2.79E-03 | 1.30567 | 1.30567 | 6.51E-04 | 0.737175 | -1.35653 |
| 10739209 | --- | 2.79E-03 | 1.30567 | 1.30567 | 6.51E-04 | 0.737175 | -1.35653 |
| 10788077 | --- | 2.79E-03 | 1.30567 | 1.30567 | 6.51E-04 | 0.737175 | -1.35653 |
| 10863402 | --- | 2.79E-03 | 1.30567 | 1.30567 | 6.51E-04 | 0.737175 | -1.35653 |
| 10885957 | --- | 2.79E-03 | 1.30567 | 1.30567 | 6.51E-04 | 0.737175 | -1.35653 |
| 10922249 | --- | 2.79E-03 | 1.30567 | 1.30567 | 6.51E-04 | 0.737175 | -1.35653 |
| 10937321 | --- | 2.79E-03 | 1.30567 | 1.30567 | 6.51E-04 | 0.737175 | -1.35653 |
| 10801135 | --- | 2.88E-03 | 2.38976 | 2.38976 | 1.37E-04 | 0.304284 | -3.2864 |
| 10889415 | Sox11 | 2.95E-03 | 1.22936 | 1.22936 | 5.73E-12 | 0.318895 | -3.13583 |
| 10847156 | Olr673 | 3.03E-03 | 1.51056 | 1.51056 | 1.37E-03 | 0.647111 | -1.54533 |
| 10700595 | --- | 3.13E-03 | 1.69696 | 1.69696 | 5.10E-03 | 0.626876 | -1.59521 |
| 10833876 | Prdm1 | 3.14E-03 | 1.17786 | 1.17786 | 1.75E-06 | 0.710599 | -1.40726 |
| 10891324 | Zdhhc22 | 3.15E-03 | 0.783558 | -1.27623 | 2.91E-05 | 1.48355 | 1.48355 |
| 10897285 | Scx | 3.15E-03 | 0.840018 | -1.19045 | 1.72E-08 | 1.70241 | 1.70241 |
| 10863857 | Isy1 | 3.16E-03 | 1.21416 | 1.21416 | 3.07E-06 | 0.67972 | -1.47119 |
| 10781829 | Klf5 | 3.18E-03 | 1.26724 | 1.26724 | 7.97E-04 | 0.763995 | -1.30891 |
| 10701108 | --- | 3.20E-03 | 1.95151 | 1.95151 | 6.27E-03 | 0.563343 | -1.77512 |
| **10858573** | **Clecsf6** | **3.28E-03** | **0.698008** | **-1.43265** | **1.62E-06** | **0.467561** | **-2.13876** |
| 10917085 | --- | 3.35E-03 | 1.20614 | 1.20614 | 7.06E-06 | 0.705366 | -1.4177 |
| 10810677 | RGD1307357 | 3.38E-03 | 0.877964 | -1.139 | 1.20E-05 | 1.25993 | 1.25993 |
| 10929761 | --- | 3.38E-03 | 1.47859 | 1.47859 | 6.46E-03 | 0.714085 | -1.40039 |
| 10718598 | --- | 3.39E-03 | 1.38574 | 1.38574 | 2.27E-04 | 0.649074 | -1.54066 |
| 10709875 | Adm | 3.40E-03 | 0.882903 | -1.13263 | 6.21E-03 | 1.11403 | 1.11403 |
| 10839417 | LOC679683 | 3.41E-03 | 1.53901 | 1.53901 | 2.67E-05 | 0.491185 | -2.03589 |
| 10773162 | --- | 3.43E-03 | 0.808324 | -1.23713 | 1.11E-03 | 1.26456 | 1.26456 |
| 10733427 | RGD1306484 | 3.47E-03 | 0.856516 | -1.16752 | 7.09E-03 | 1.141 | 1.141 |
| 10771267 | Mapk10 | 3.49E-03 | 0.749858 | -1.33358 | 4.81E-06 | 1.7473 | 1.7473 |
| 10884994 | Nin | 3.55E-03 | 0.747648 | -1.33753 | 3.16E-04 | 1.45349 | 1.45349 |
| 10746899 | --- | 3.60E-03 | 1.36002 | 1.36002 | 3.87E-04 | 0.679004 | -1.47274 |
| 10869541 | --- | 3.63E-03 | 0.823604 | -1.21418 | 2.48E-03 | 1.21443 | 1.21443 |
| 10797013 | --- | 3.71E-03 | 2.50316 | 2.50316 | 3.03E-04 | 0.303357 | -3.29644 |
| 10932228 | --- | 3.71E-03 | 2.50316 | 2.50316 | 3.03E-04 | 0.303357 | -3.29644 |
| 10844082 | Ntng2 | 3.74E-03 | 0.872542 | -1.14608 | 3.02E-06 | 1.32082 | 1.32082 |
| 10771984 | Ppia | 3.76E-03 | 0.903699 | -1.10656 | 6.79E-03 | 1.09181 | 1.09181 |
| 10867045 | Ppia | 3.76E-03 | 0.903699 | -1.10656 | 6.79E-03 | 1.09181 | 1.09181 |
| 10746776 | --- | 3.81E-03 | 0.846191 | -1.18177 | 4.91E-03 | 1.16484 | 1.16484 |
| 10840353 | Banf2 | 3.84E-03 | 0.87046 | -1.14882 | 2.18E-03 | 1.15316 | 1.15316 |
| 10878965 | --- | 3.90E-03 | 1.57639 | 1.57639 | 3.30E-05 | 0.472362 | -2.11702 |
| 10834662 | RGD1311501 | 3.91E-03 | 1.10263 | 1.10263 | 5.52E-04 | 0.887361 | -1.12694 |
| 10828884 | Pim1 | 3.91E-03 | 0.852582 | -1.17291 | 1.75E-10 | 0.490203 | -2.03997 |
| 10834602 | --- | 4.13E-03 | 1.99266 | 1.99266 | 4.26E-05 | 0.327245 | -3.05581 |
| 10848416 | Fsip1 | 4.14E-03 | 1.23425 | 1.23425 | 9.02E-03 | 0.837969 | -1.19336 |
| 10703618 | Vom2r21 | 4.15E-03 | 1.1772 | 1.1772 | 3.86E-04 | 0.810766 | -1.2334 |
| 10797766 | Fam8a1 | 4.17E-03 | 0.824712 | -1.21254 | 8.82E-05 | 1.33734 | 1.33734 |
| 10700004 | --- | 4.20E-03 | 1.11312 | 1.11312 | 3.72E-04 | 0.870547 | -1.1487 |
| 10780446 | Rec8 | 4.29E-03 | 0.890299 | -1.12322 | 1.26E-03 | 1.13922 | 1.13922 |
| 10734720 | Pik3r5 | 4.29E-03 | 0.735108 | -1.36034 | 4.47E-04 | 0.676358 | -1.47851 |
| 10935047 | Ngfrap1 | 4.29E-03 | 0.855831 | -1.16845 | 4.16E-03 | 1.15996 | 1.15996 |
| 10874210 | Uts2 | 4.32E-03 | 1.5809 | 1.5809 | 4.09E-06 | 0.394803 | -2.53291 |
| 10745335 | Omg | 4.34E-03 | 0.88208 | -1.13368 | 3.49E-07 | 1.3697 | 1.3697 |
| 10706216 | Tshz3 | 4.39E-03 | 0.838422 | -1.19272 | 2.42E-07 | 1.57824 | 1.57824 |
| 10773298 | Mrfap1 | 4.44E-03 | 1.10624 | 1.10624 | 4.93E-06 | 0.816842 | -1.22423 |
| 10837366 | Slc43a3 | 4.56E-03 | 1.35818 | 1.35818 | 1.64E-07 | 0.439123 | -2.27727 |
| 10889036 | Kcnk3 | 4.60E-03 | 0.783523 | -1.27629 | 2.69E-05 | 1.5219 | 1.5219 |
| 10867364 | --- | 4.65E-03 | 0.820861 | -1.21823 | 4.84E-03 | 1.2046 | 1.2046 |
| 10734338 | --- | 4.77E-03 | 1.24958 | 1.24958 | 5.59E-05 | 0.698346 | -1.43195 |
| 10840245 | Snap25 | 4.87E-03 | 0.769526 | -1.2995 | 2.05E-05 | 1.59501 | 1.59501 |
| 10703222 | --- | 4.87E-03 | 1.17361 | 1.17361 | 5.28E-04 | 0.816012 | -1.22547 |
| 10759597 | --- | 4.92E-03 | 1.33793 | 1.33793 | 1.55E-03 | 0.72291 | -1.3833 |
| 10722241 | Csrp3 | 4.93E-03 | 1.68941 | 1.68941 | 3.84E-09 | 0.146748 | -6.81439 |
| 10845743 | Fign | 4.94E-03 | 0.786427 | -1.27157 | 2.38E-05 | 1.52643 | 1.52643 |
| 10779309 | Spetex-2E | 5.07E-03 | 1.99414 | 1.99414 | 4.73E-03 | 0.515933 | -1.93824 |
| 10759492 | Gapdh | 5.08E-03 | 0.947676 | -1.05521 | 1.94E-03 | 1.06016 | 1.06016 |
| 10851587 | Matn4 | 5.24E-03 | 1.19273 | 1.19273 | 7.31E-04 | 0.804253 | -1.24339 |
| 10861986 | Insig1 | 5.32E-03 | 0.862847 | -1.15895 | 8.18E-07 | 1.42634 | 1.42634 |
| 10886494 | Ubr7 | 5.34E-03 | 1.17599 | 1.17599 | 2.68E-04 | 0.798157 | -1.25289 |
| 10814498 | LOC499584 | 5.35E-03 | 1.15036 | 1.15036 | 1.13E-04 | 0.807566 | -1.23829 |
| 10904528 | Lynx1 | 5.41E-03 | 0.842716 | -1.18664 | 3.27E-04 | 1.26281 | 1.26281 |
| 10877978 | RGD1564517 | 5.54E-03 | 0.74003 | -1.3513 | 1.19E-03 | 1.42379 | 1.42379 |
| 10866709 | Ldhb | 5.58E-03 | 0.893695 | -1.11895 | 7.14E-04 | 1.15101 | 1.15101 |
| 10884078 | --- | 5.60E-03 | 1.21022 | 1.21022 | 6.49E-03 | 0.837856 | -1.19352 |
| 10781146 | Pbk | 5.61E-03 | 1.25214 | 1.25214 | 9.96E-14 | 0.159036 | -6.28787 |
| 10809018 | Cmtm3 | 5.62E-03 | 1.22056 | 1.22056 | 1.18E-11 | 0.316693 | -3.15763 |
| 10770706 | Fam71a | 5.73E-03 | 0.802336 | -1.24636 | 1.93E-03 | 1.27655 | 1.27655 |
| 10727734 | Rab1b | 5.75E-03 | 1.23576 | 1.23576 | 1.87E-05 | 0.676855 | -1.47742 |
| 10853149 | --- | 5.77E-03 | 0.783956 | -1.27558 | 5.68E-06 | 1.65064 | 1.65064 |
| 10700064 | --- | 5.84E-03 | 1.40861 | 1.40861 | 6.75E-04 | 0.64745 | -1.54452 |
| 10701611 | --- | 5.89E-03 | 1.28831 | 1.28831 | 1.93E-04 | 0.690108 | -1.44905 |
| 10872059 | RGD1563072 | 5.93E-03 | 0.869818 | -1.14967 | 4.49E-06 | 1.34239 | 1.34239 |
| 10838282 | --- | 5.96E-03 | 2.65937 | 2.65937 | 1.49E-03 | 0.323414 | -3.09201 |
| 10924172 | --- | 5.96E-03 | 2.65937 | 2.65937 | 1.49E-03 | 0.323414 | -3.09201 |
| 10734316 | Ubb | 5.98E-03 | 0.929698 | -1.07562 | 8.91E-03 | 1.06701 | 1.06701 |
| 10783667 | Ppp1r3e | 6.02E-03 | 0.827862 | -1.20793 | 5.42E-03 | 1.19976 | 1.19976 |
| 10749922 | --- | 6.21E-03 | 0.832355 | -1.20141 | 9.30E-04 | 1.25371 | 1.25371 |
| 10779031 | Camk2g | 6.27E-03 | 0.875878 | -1.14171 | 1.20E-07 | 1.46751 | 1.46751 |
| 10728949 | Plac1l | 6.34E-03 | 0.851465 | -1.17445 | 5.83E-03 | 1.16711 | 1.16711 |
| 10921617 | Ttbk1 | 6.41E-03 | 0.839926 | -1.19058 | 1.48E-04 | 1.30489 | 1.30489 |
| 10911818 | Cd109 | 6.41E-03 | 1.25367 | 1.25367 | 1.95E-06 | 0.594578 | -1.68186 |
| 10880738 | Epha8 | 6.48E-03 | 0.780668 | -1.28095 | 6.63E-04 | 1.37707 | 1.37707 |
| **10865993** | **Cd69** | **6.52E-03** | **1.1846** | **1.1846** | **1.82E-05** | **0.726595** | **-1.37628** |
| 10755770 | Tmem191c | 6.59E-03 | 0.883988 | -1.13124 | 1.86E-04 | 1.20251 | 1.20251 |
| 10735747 | --- | 6.75E-03 | 1.15932 | 1.15932 | 2.88E-03 | 0.852865 | -1.17252 |
| 10922068 | Crisp1 | 6.78E-03 | 1.43438 | 1.43438 | 2.04E-08 | 0.295874 | -3.37982 |
| 10884574 | --- | 6.78E-03 | 0.778434 | -1.28463 | 8.96E-04 | 1.36938 | 1.36938 |
| 10812346 | Cetn3 | 6.82E-03 | 1.1794 | 1.1794 | 5.40E-04 | 0.802318 | -1.24639 |
| 10882461 | RGD1562284 | 6.86E-03 | 0.814328 | -1.22801 | 5.32E-07 | 1.70469 | 1.70469 |
| 10928976 | --- | 6.92E-03 | 0.84064 | -1.18957 | 1.12E-03 | 1.23699 | 1.23699 |
| 10865186 | Ccdc77 | 7.01E-03 | 1.21251 | 1.21251 | 2.17E-04 | 0.7511 | -1.33138 |
| 10701058 | --- | 7.01E-03 | 0.805939 | -1.24079 | 2.05E-04 | 1.38066 | 1.38066 |
| 10701307 | --- | 7.06E-03 | 0.876318 | -1.14114 | 1.23E-03 | 1.17382 | 1.17382 |
| 10929153 | Resp18 | 7.06E-03 | 0.787446 | -1.26993 | 3.74E-05 | 1.53065 | 1.53065 |
| 10880539 | Man1c1 | 7.13E-03 | 0.809531 | -1.23528 | 4.28E-05 | 0.689326 | -1.45069 |
| 10894432 | --- | 7.20E-03 | 1.14413 | 1.14413 | 7.71E-04 | 0.840521 | -1.18974 |
| 10846928 | --- | 7.35E-03 | 1.76362 | 1.76362 | 1.83E-03 | 0.517476 | -1.93246 |
| 10777726 | RGD1560394 | 7.37E-03 | 0.801295 | -1.24798 | 2.69E-04 | 1.38257 | 1.38257 |
| 10700027 | --- | 7.39E-03 | 1.26954 | 1.26954 | 5.27E-04 | 0.723547 | -1.38208 |
| 10923877 | Adam23 | 7.45E-03 | 0.820706 | -1.21846 | 8.22E-05 | 1.38851 | 1.38851 |
| 10892305 | LOC691427 | 7.46E-03 | 0.784445 | -1.27479 | 5.09E-03 | 1.27697 | 1.27697 |
| 10845407 | Cytip | 7.48E-03 | 1.26036 | 1.26036 | 8.97E-07 | 0.558207 | -1.79145 |
| 10895020 | --- | 7.56E-03 | 1.20713 | 1.20713 | 2.32E-03 | 0.808491 | -1.23687 |
| 10918342 | Fbxl22 | 7.57E-03 | 0.799447 | -1.25086 | 1.34E-03 | 1.31231 | 1.31231 |
| 10909141 | Olr1219 | 7.61E-03 | 1.18928 | 1.18928 | 1.22E-03 | 0.807898 | -1.23778 |
| 10934579 | Cox7b | 7.68E-03 | 0.801513 | -1.24764 | 6.48E-04 | 1.34256 | 1.34256 |
| 10786532 | Mustn1 | 7.69E-03 | 1.42761 | 1.42761 | 2.59E-09 | 0.236999 | -4.21943 |
| 10801691 | --- | 7.71E-03 | 1.12129 | 1.12129 | 6.19E-03 | 0.893613 | -1.11905 |
| 10917905 | Cd276 | 7.72E-03 | 1.1492 | 1.1492 | 2.04E-06 | 0.720263 | -1.38838 |
| 10706181 | Slc7a9 | 7.76E-03 | 1.19681 | 1.19681 | 1.13E-03 | 0.79944 | -1.25088 |
| 10878813 | --- | 7.85E-03 | 0.800619 | -1.24903 | 2.57E-03 | 1.28296 | 1.28296 |
| 10934239 | Kif4 | 7.88E-03 | 1.20834 | 1.20834 | 1.15E-09 | 0.441501 | -2.265 |
| 10851219 | Ndrg3 | 8.00E-03 | 0.863125 | -1.15858 | 6.00E-05 | 1.29094 | 1.29094 |
| 10879688 | --- | 8.04E-03 | 1.21661 | 1.21661 | 5.16E-03 | 0.819213 | -1.22068 |
| 10863874 | H1fx | 8.06E-03 | 0.909806 | -1.09914 | 8.68E-03 | 1.09263 | 1.09263 |
| 10815882 | --- | 8.10E-03 | 0.811233 | -1.23269 | 3.46E-03 | 1.25355 | 1.25355 |
| 10807044 | Arhgap10 | 8.12E-03 | 1.38966 | 1.38966 | 2.60E-08 | 0.325653 | -3.07075 |
| 10844275 | Fibcd1 | 8.21E-03 | 0.757598 | -1.31996 | 4.63E-03 | 1.33378 | 1.33378 |
| 10809088 | --- | 8.23E-03 | 0.79812 | -1.25295 | 1.01E-03 | 1.33333 | 1.33333 |
| 10752758 | Btg3 | 8.25E-03 | 1.20255 | 1.20255 | 2.81E-05 | 0.707228 | -1.41397 |
| 10846821 | Tmx2 | 8.41E-03 | 0.883891 | -1.13136 | 4.04E-03 | 1.1402 | 1.1402 |
| 10930598 | --- | 8.41E-03 | 0.929544 | -1.0758 | 1.26E-07 | 1.24702 | 1.24702 |
| 10895499 | Tspan8 | 8.46E-03 | 1.43642 | 1.43642 | 9.33E-07 | 0.395415 | -2.52899 |
| 10798163 | LOC364707 | 8.48E-03 | 0.895173 | -1.1171 | 7.25E-03 | 1.1138 | 1.1138 |
| 10871769 | --- | 8.50E-03 | 1.89412 | 1.89412 | 2.93E-03 | 0.490624 | -2.03822 |
| 10700550 | --- | 8.58E-03 | 1.71728 | 1.71728 | 7.56E-04 | 0.486954 | -2.05358 |
| 10708095 | LOC691427 | 8.58E-03 | 0.782099 | -1.27861 | 6.50E-03 | 1.2761 | 1.2761 |
| 10725039 | LOC691427 | 8.58E-03 | 0.782099 | -1.27861 | 6.50E-03 | 1.2761 | 1.2761 |
| 10734815 | Rnf222 | 8.59E-03 | 0.773155 | -1.2934 | 9.36E-03 | 1.27207 | 1.27207 |
| 10770858 | Cr2 | 8.60E-03 | 1.51358 | 1.51358 | 1.22E-05 | 0.428268 | -2.33498 |
| 10778080 | --- | 8.60E-03 | 2.02865 | 2.02865 | 1.10E-03 | 0.4068 | -2.45821 |
| 10704858 | Nkpd1 | 8.60E-03 | 0.796247 | -1.25589 | 1.58E-03 | 1.31876 | 1.31876 |
| 10815701 | --- | 8.61E-03 | 1.18852 | 1.18852 | 1.64E-03 | 0.811629 | -1.23209 |
| 10846248 | Atp5g3 | 8.64E-03 | 0.872978 | -1.1455 | 5.39E-04 | 1.20731 | 1.20731 |
| 10886599 | --- | 8.82E-03 | 1.30271 | 1.30271 | 2.86E-08 | 0.403774 | -2.47663 |
| 10759511 | Pabpc4 | 8.85E-03 | 1.31247 | 1.31247 | 2.44E-03 | 0.731187 | -1.36764 |
| 10817233 | Lingo4 | 8.96E-03 | 0.833221 | -1.20016 | 5.06E-07 | 1.64357 | 1.64357 |
| 10883159 | Ucn | 8.96E-03 | 1.54984 | 1.54984 | 4.40E-10 | 0.125035 | -7.99775 |
| 10844598 | Phf19 | 8.97E-03 | 1.21509 | 1.21509 | 4.93E-03 | 0.816105 | -1.22533 |
| 10716500 | RGD1563099 | 9.00E-03 | 0.834783 | -1.19792 | 1.01E-03 | 1.26346 | 1.26346 |
| 10749716 | Pycr1 | 9.14E-03 | 0.82808 | -1.20761 | 3.60E-04 | 1.31873 | 1.31873 |
| 10869728 | Sh3gl2 | 9.23E-03 | 0.794233 | -1.25908 | 4.73E-04 | 1.38826 | 1.38826 |
| 10701348 | --- | 9.32E-03 | 0.886341 | -1.12823 | 3.50E-05 | 1.25406 | 1.25406 |
| 10886806 | Dlk1 | 9.32E-03 | 0.813753 | -1.22887 | 3.06E-03 | 1.26101 | 1.26101 |
| 10925449 | Twist2 | 9.35E-03 | 0.73502 | -1.36051 | 3.17E-03 | 1.4118 | 1.4118 |
| 10845859 | Scn1a | 9.40E-03 | 0.822254 | -1.21617 | 7.43E-07 | 1.68226 | 1.68226 |
| 10846916 | --- | 9.45E-03 | 0.929518 | -1.07583 | 3.07E-03 | 1.08587 | 1.08587 |
| 10833093 | H2afy2 | 9.45E-03 | 0.840539 | -1.18971 | 1.33E-05 | 1.43059 | 1.43059 |
| 10862795 | Nap1l5 | 9.45E-03 | 0.866778 | -1.1537 | 1.51E-06 | 1.43052 | 1.43052 |
| 10757599 | Ywhag | 9.48E-03 | 0.874794 | -1.14313 | 1.53E-03 | 1.18027 | 1.18027 |
| 10706236 | Uqcrb | 9.49E-03 | 0.888469 | -1.12553 | 4.72E-05 | 1.24128 | 1.24128 |
| 10818245 | Kcna2 | 9.63E-03 | 0.80057 | -1.24911 | 8.80E-05 | 1.46639 | 1.46639 |
| 10919534 | RGD1564597 | 9.69E-03 | 1.47547 | 1.47547 | 9.14E-03 | 0.688935 | -1.45152 |
| 10772768 | Tlr6 | 9.72E-03 | 1.22951 | 1.22951 | 3.22E-07 | 0.553015 | -1.80827 |
| 10740959 | Kctd5 | 9.82E-03 | 1.16052 | 1.16052 | 4.67E-03 | 0.853056 | -1.17226 |
| 10937800 | --- | 9.86E-03 | 1.33351 | 1.33351 | 3.80E-03 | 0.728347 | -1.37297 |
| 10802980 | Mbp | 9.91E-03 | 0.892957 | -1.11987 | 2.77E-06 | 1.31142 | 1.31142 |
| 10877921 | Slc24a2 | 9.93E-03 | 0.813247 | -1.22964 | 9.80E-04 | 1.31411 | 1.31411 |
| 10842298 | Eya2 | 9.94E-03 | 1.16724 | 1.16724 | 2.09E-05 | 0.734758 | -1.36099 |

TheC-type lectin genes Clec4a3/Dcir3, Clec4a2/Dcir2, Clecsf6/Dcir1 and Cd69 are marked in bold and underlined. All genes that differed between operated Aplec and DA rats also differed significantly between operated and naïve Aplec rats demonstrating that they all were a part of the differentially regulated injury response.
