# Supplementary figures and images for: Genetic variability in the rat Aplec C-type lectin gene cluster regulates lymphocyte trafficking and motor neuron survival after traumatic nerve root injury
Source: J Neuroinflammation. 2013 May 8;10:60. doi: 10.1186/1742-2094-10-60 (PMC3661385; doi:10.1186/1742-2094-10-60)

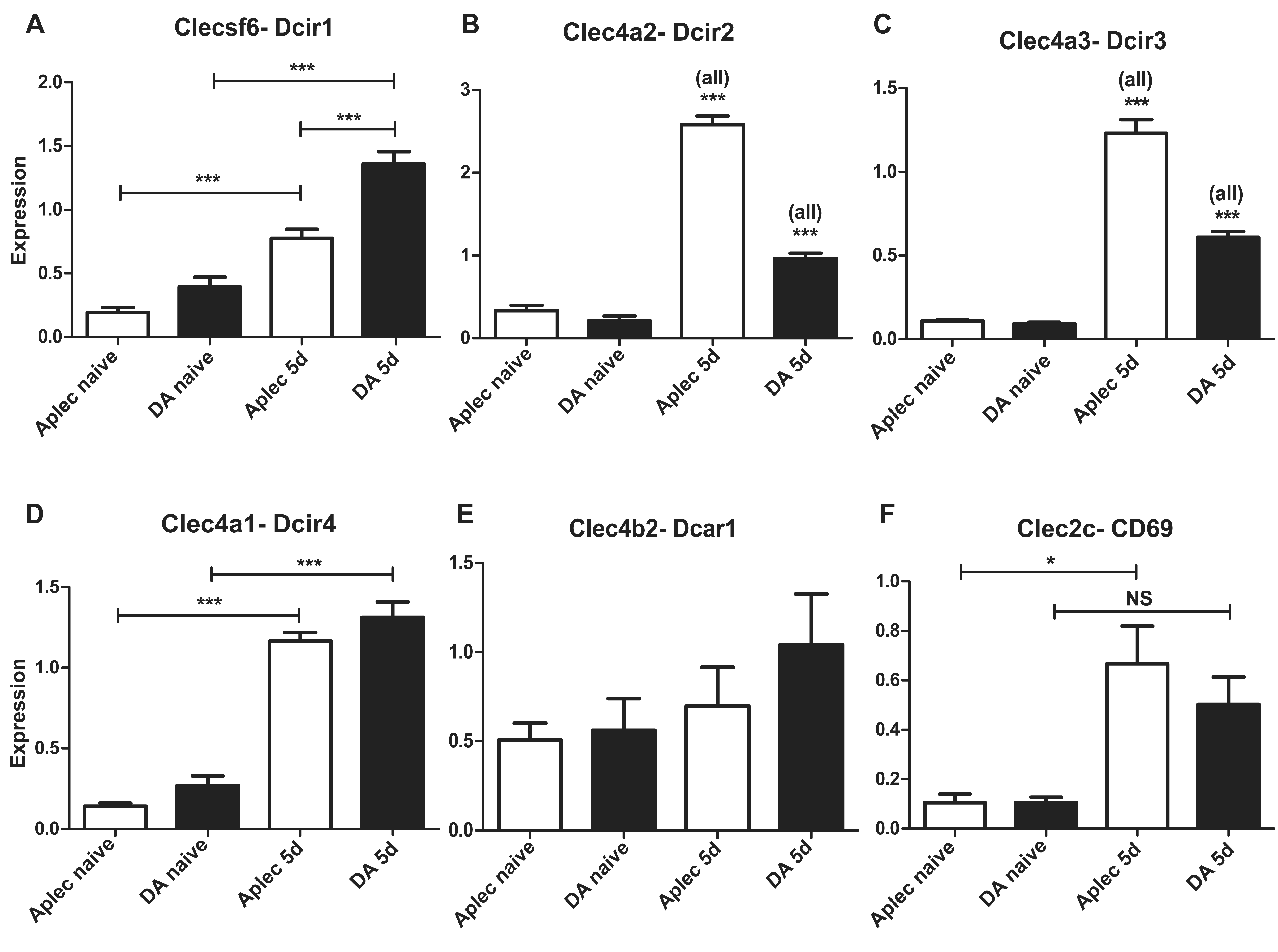

Supplement: Additional file 2: Figure S1 — RT-PCR confirmation of the expression of the Aplec genes and Cd69. To confirm the results from the microarray expressional profiling of the DA and Aplec spinal cords, RT-PCR analysis of expression in the L3 segments from naïve and VRA operated animals from both strains was performed. The RT-PCR quantification confirmed the microarray results; Clecsf6 (Dcir1), Clec4a2 (Dcir2), Clec4a3 (Dcir3) and Clec4a1(Dcir4), were all upregulated following injury in both strains, but with higher expression of Clec4a2 and Clec4a3 in Aplec and of Clecsf6 in DA and no strain differences in Clec4a1 (A-D). Dcar showed no injury or strain variation (E). Cd69 was significantly upregulated only in the Aplec strain (F). [file 1742-2094-10-60-S2.tiff]

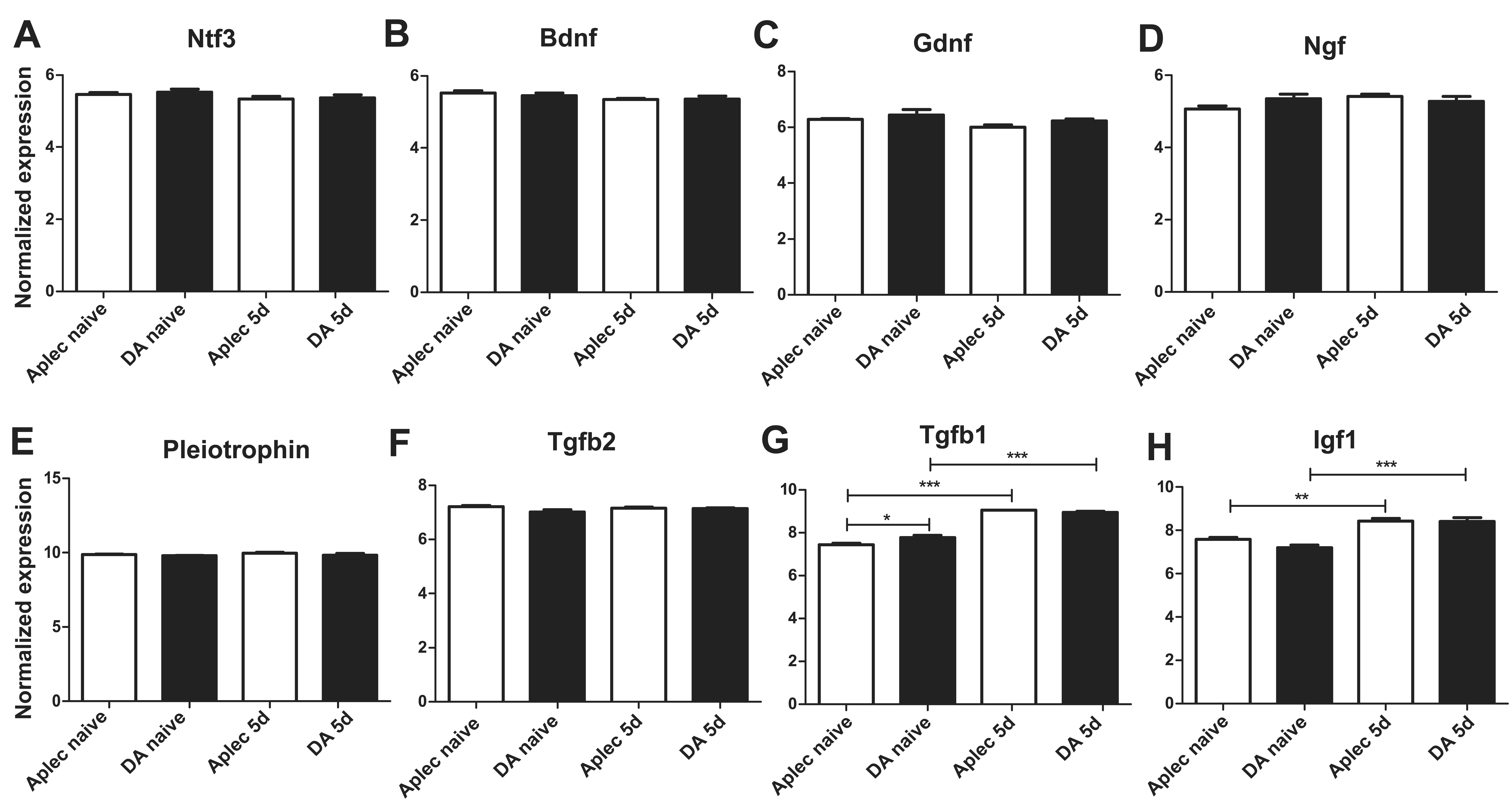

Supplement: Additional file 3: Figure S2 — Expression of neurotrophic factors in DA and Aplec rats. Expression of the most acknowledged factors with neurotrophic effects was assessed in the microarray expressional profiling data set. Expression of (A) neurotrophin-3 (Ntf3), (B) brain-derived neurotrophic factor (Bdnf), (C) glial-derived neurotrophic factor (Gdnf), (D) nerve growth factor (Ngf), (E) pleiotrophin and (F) transforming growth factor β2 (Tgfb2), was regulated by neither injury nor strain. In contrast, the expression of (G) transforming growth factor β1 (Tgfb1) and (H) insulin growth factor 1 (Igf1) was upregulated in both strains, but without differences. [file 1742-2094-10-60-S3.tiff]
